# International Conference on Translational Medicine 2025 Abstracts

**DOI:** 10.1515/jtim-2025-0061

**Published:** 2025-12-22

This issue of the Journal of Translational Internal Medicine features abstracts from the International Conference on Translational Medicine 2025 (ICTM2025). The conference, held under the theme of "Innovative Transformation," took place from October 17–19, 2025 in Beijing.

The purpose of this conference was to establish a diversified exchange platform for clinicians, medical researchers, innovative pharmaceutical enterprises, and other stakeholders. The platform covered areas such as multidisciplinary collaborative diagnosis and treatment, clinical innovation practice, translational medicine research, medical artificial intelligence, integration of big data and advanced technologies, and transformation of innovative technologies.

To enhance the readability of this special feature, abstracts have been edited for basic style only. The content has not been modified; the information provided reflects information that was submitted by the primary author, including professional degrees and affiliations.

Website: www.intern-med.com

## Analysis of entecavir resistance mutation evolution and clinical implications over the past five years

**Xuyang Li^1^, Lanlan Si^2^, Le Li^2^, Mengwen He^1^, Xueyuan Chen^2^, Zengtao Yao^2^, Chunyan Wang^3^, Dong Ji^1,3^, Yan Liu^2^**

^1^Peking University 302 Clinical Medical School, Beijing, China;

^2^Senior Department of Infectious Diseases, the Fifth Medical Center of PLA General Hospital, Beijing, China;

^3^Senior Department of Hepatology, the Fifth Medical Center of PLA General Hospital, Beijing, China

Address for Correspondence: Yan Liu, Email: liuyan5360@163.com; Dong Ji, Email: jidg302@126.com

### Abstract

**Background and Objectives**: To analyze the characteristics of entecavir (ETV) genotypic resistance mutations in patients with chronic hepatitis B virus (HBV) infection over the past five years, aiming to elucidate the evolving patterns of ETV resistance and provide a basis for rational clinical antiviral therapy. **Methods**: This study included 4697 chronic HBV-infected patients who underwent HBV resistance mutation testing at the Fifth Medical Center of PLA General Hospital between July 2019 and July 2024. All patients had received nucleos(t)ide analogs (NAs) therapy. Viral genomes were extracted from serum, and the HBV reverse transcriptase (RT) gene region was amplified using single-tube nested PCR, followed by Sanger sequencing to analyze NAs resistance associated mutations. Clinical data were combined with logistic regression analysis to identify risk factors for ETV resistance. **Results**: ETV resistance mutations were detected in 547 (11.6%) of the 4697 patients. Resistance mutations were predominantly observed in patients with prior NAs therapy (Figure A), primarily those treated with lamivudine (LAM)→ETV (66.5%) or LAM→adefovir dipivoxil (ADV)→ETV sequential therapy (20.0%). Among the 59 detected ETV resistance mutation patterns, mutations based on the rtS202 site were the most common (302 cases, 55.2%), followed by rtT184 site mutations (233 cases, 42.6%), and rtM250 site mutations (71 cases, 13.0%). Compared to the non-ETV-mutated group, the ETV resistance group had significantly higher proportions of males, older age, elevated ALT levels, higher HBV DNA loads, HBeAg positivity, HBV genotype C, cirrhosis, and a history of LAM therapy (*P* < 0.05). Multivariable analysis identified age ≥ 45 years, HBeAg positivity, HBV DNA ≥ 3.0 log_10_ IU/mL, HBV genotype C, cirrhosis, and prior LAM therapy as independent risk factors for ETV resistance. **Conclusion**: As a first-line potent oral nucleoside antiviral drug recommended by current guidelines, the clinical pattern of ETV resistance is evolving, with the rtS202G+rtL180M+rtM204V mutation emerging as the predominant resistance profile. Older age, HBeAg positivity, high HBV DNA load, HBV genotype C, cirrhosis, and prior LAM therapy are key risk factors. Clinicians should remain vigilant of these high-risk factors and exercise caution when considering ETV for rescue therapy.

**How to cite this abstract**: Li X, Si L, Li L, He M, Chen X, Yao Z, *et al*. Analysis of entecavir resistance mutation evolution and clinical implications over the past five years. J Transl Intern Med 2025; 13: A623-A624.

**Figure:**
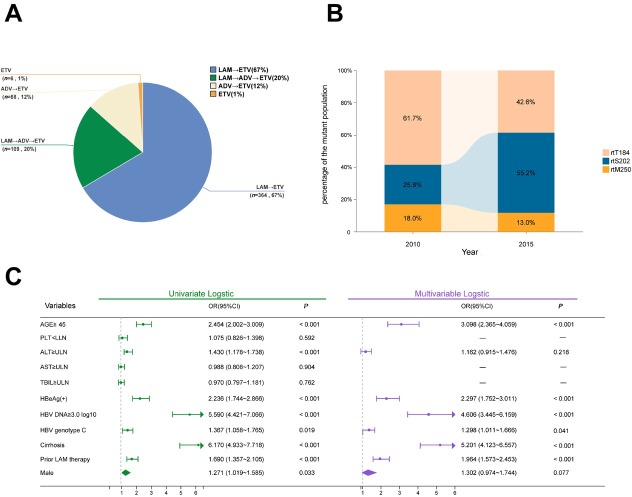
(A) NAs treatment approaches for patients with ETV resistance mutations. (B) Changes in RT mutation patterns over years. (C) Forest plot of risk factors for HBV entecavir resistance. NAs: nucleos(t)ide analogs; LAM: lamivudine; ETV: entecavir; ADV: adefovir dipivoxil; HBV: hepatitis B virus. PLT, platelet; ALT, alanine aminotransferase; AST, aspartate aminotransferase; TBIL, total bilirubin; OR, odds ratio; CI, confidence interval.

## Clinical efficacy observation of particle implantation therapy for hepatocellular carcinoma and extrahepatic metastases

**Chuang Li, Yunfeng Kou, Zhongyi Ding, Weichang Li, Zhuo Wang**

The Affiliated Zhongshan Hospital of DalianUniversity, Dalian, Liaoning Province, China

Address for Correspondence: Chuan Li, Email: lichuang1001@163.com

### Abstract

**Background and Objectives**: This study aims to evaluate the clinical efficacy and safety of CT-guided ^125^| radioactive seed implantation in the treatment of hepatocellular carcinoma and extrahepatic metastases. **Methods**: Clinical data of 31 patients with hepatocellular carcinoma and extrahepatic metastases who underwent CT-guided ^125^| radioactive seed implantation from January 2020 to December 2023 were collected. The disease control rate (DCR), objective response rate (ORR), overall survival (OS), pain relief rate, quality of life score, and surgical complications were evaluated. Additionally, preliminary analysis was performed on the changes in peripheral blood lymphocyte subsets and the expression of some immune factors at 1, 3, and 6 months after surgery. **Results**: The median follow-up time was 11.8 months (range, 6–18 months). The 6-month DCR was 79.25%. The ORR at 1, 3, and 6 months was 32.08%, 58.41%, and 62.36%, respectively. The 12-month and 24-month OS rates were 74.68% and 30.12%, while the 12-month and 24-month progression-free survival (PFS) rates were 60.38% and 32.36%, respectively. The postoperative pain relief rate was 85.79%. No severe complications such as massive hemorrhage, severe infection, or organ perforation were observed. In some patients, the levels of CD3^+^CD4^+^ T cells and CD3^+^CD8^+^ T cells in peripheral blood at 3 and 6 months after surgery were significantly higher than those before surgery and at 1 month after surgery (*P* < 0.01). Additionally, the natural killer (NK) cell levels at 1 and 3 months after surgery were significantly higher than those before surgery (*P* < 0.01). **Conclusion**: CT-guided ^125^| radioactive seed implantation for hepatocellular carcinoma and extrahepatic metastases is minimally invasive, which can effectively improve patients' quality of life, reduce tumor burden, and induce positive immune changes in the body.

**How to cite this abstract**: Li C, Kou Y, Ding Z, Li W, Wang Z. Clinical efficacy observation of particle implantation therapy for hepatocellular carcinoma and extrahepatic metastases. J Transl Intern Med 2025; 13: A625.

## Orlistat-dopamine conjugate micelles improve targeted delivery and therapeutic efficiency of camptothecin in combination chemotherapy

**Xiaqing Zhou^1^, Haoyu Wang^2^, Shuangsuo Dang^1^**

^1^Department of Infectious Diseases, Second Affiliated Hospital of Xi'an Jiaotong University, Xi'an, Shaanxi Province, China;

^2^Bioinspired Engineering and Biomechanics Center (BEBC), Xi’an Jiaotong University, Xi’an, Shaanxi Province, China

Address for Correspondence: Shuangsuo Dang, Email: dang212@126.com

### Abstract

**Background and Objectives**: Monotherapies in cancer treatment often face limitations such as drug resistance, systemic toxicity, and inadequate tumor targeting—especially under hypoxic conditions. To address these challenges, we developed a novel nanocarrier-based combination therapy leveraging a dual-action platform: orlistat-dopamine conjugate micelles (ODCM) encapsulating camptothecin (CPT), aimed at overcoming hypoxia-induced resistance and enhancing synergistic antitumor efficacy. **Methods**: ODCMs were synthesized *via* Schiff-base reaction and characterized by fourier transform infrared spectroscopy (FTIR), dynamic light scattering (DLS), and cryo-Transmission electron microscope (cryo-TEM). CPT-ODCMs were fabricated using an oil-in-water emulsion and evaluated for size, drug encapsulation efficiency (DEE), and drug loading capacity (DLC). Cellular uptake, pH-responsive CPT release, cytotoxicity under normoxia and hypoxia, and intracellular ROS generation were studied *in vitro* using breast, lung, and prostate cancer lines. MAO-A/B inhibition assays and LIVE/DEAD imaging were used to elucidate the mechanistic contribution of oxidative stress. *In vivo* antitumor efficacy was assessed in MDA-MB-231 xenograft nude mice treated intravenously with CPT-ODCMs. **Results**: CPT-ODCMs formed uniform micelles (about 150 nm, PDI about 0.26) with high DEE (92.8%) and DLC (89.2%). The micelles exhibited pH-sensitive release, with about 100% CPT released in acidic (pH 6.0) conditions within 36 h, compared to < 70% at pH7.4. Enhanced cellular uptake and selective cytotoxicity under hypoxia were observed, with a 1315-fold reduction in IC_50_ for MDA-MB-231 cells versus free CPT. Mechanistic studies revealed a two-stage action: dopamine-triggered ROS and MAO-B-mediated cytotoxicity induced early-stage lysis, followed by CPT-induced apoptosis. MAO-B inhibition significantly attenuated early toxicity, validating its mechanistic role. *In vivo*, CPT-ODCM treatment achieved about 6-fold tumor reduction versus free CPT, with no observable systemic toxicity. **Conclusion**: CPT-ODCMs provide a dual-stage therapeutic strategy that integrates fatty acid synthase inhibition, dopamine-induced oxidative stress, and CPT-mediated apoptosis, effectively targeting hypoxia-adapted, drug-resistant tumors. This programmable nanoplatform offers promising translational potential for combination cancer therapy with improved efficacy and safety.

**Figure 1:**
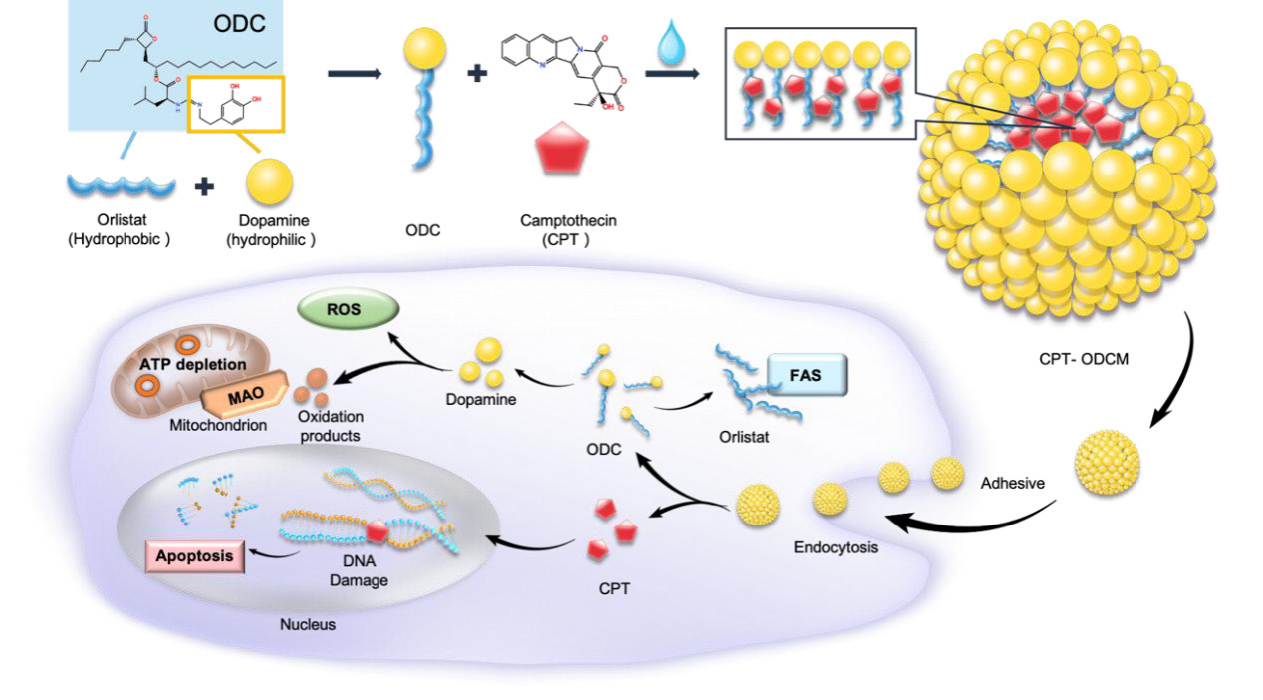
The schematic image of CPT-ODCM fabrication. CPT: camptothecin; ODCM: orlistat-dopamine conjugate micelles.

**How to cite this abstract**: Zhou X, Wang H, Dang S. Orlistat-dopamine conjugate micelles improve targeted delivery and therapeutic efficiency of camptothecin in combination chemotherapy. J Transl Intern Med 2025; 13: A626-A627.

**Figure 2:**
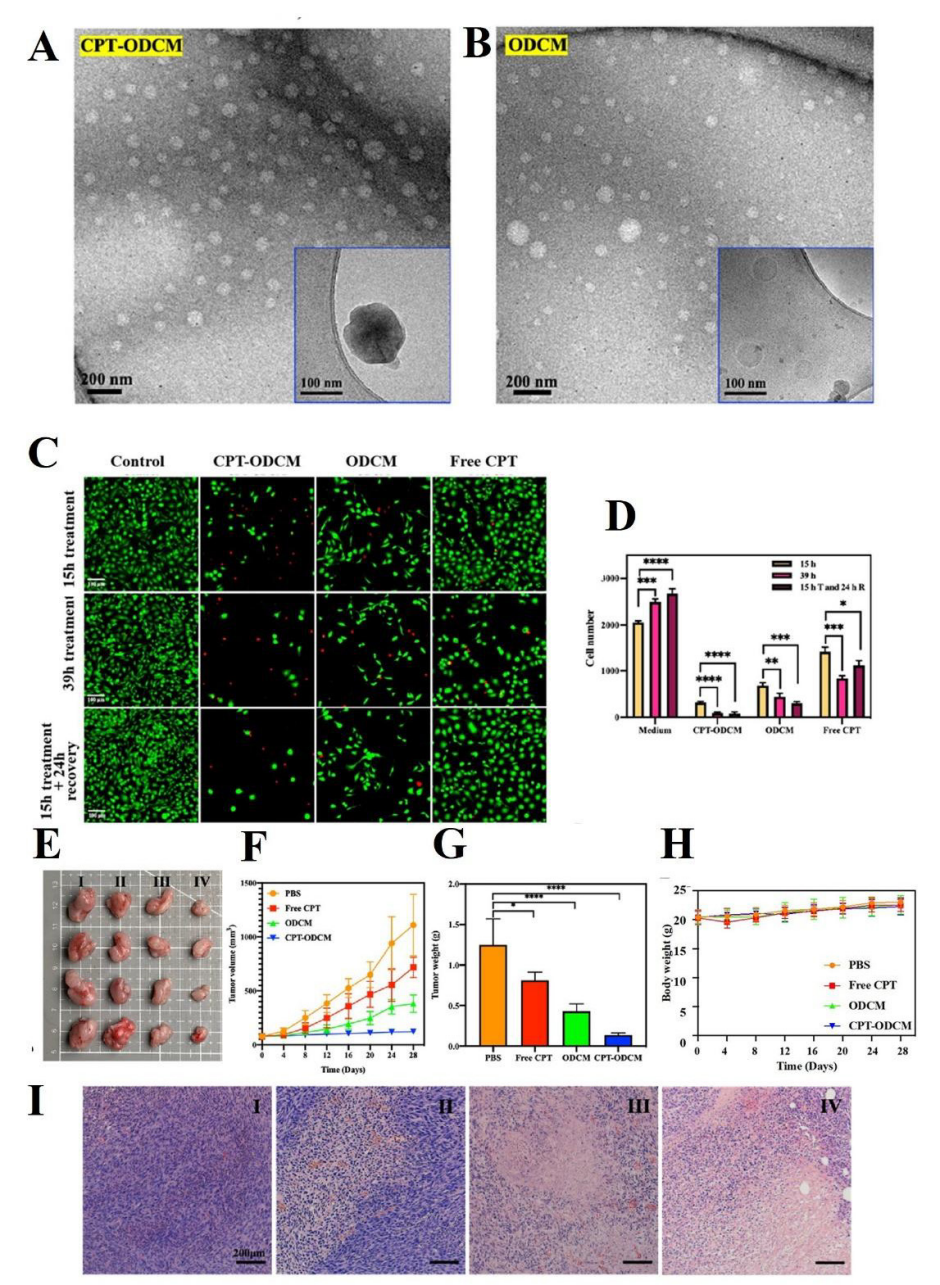
Negative stained TEM image and cryo-TEM image of CPT-ODCM (A) and ODCMs (B) (inset image in the corner). (C) fluorescence images of LIVE/ DEAD assay of MDA-MB-231 cell cultured with 0.1 mM CPT-ODCMs, ODCMs, or free CPT at different time points (15, 39 h different drug treatment, or 15 h different drug treatment with 24 h recovery in culture medium). (D) the total cell (live cells and dead cells) number summary of anti-proliferation and cell death test at different time points (15, 39 h different drug treatment, or 15 h different drug treatment with 24 h recovery in culture medium). (E) optical images of tumors with different treatments (PBS [control group, I], Free CPT [5 mg/kg, II], ODCM [90 mg/kg, III], and CPT-ODCM [95 mg/kg, equals to 5 mg CPT/kg, IV]). (F) time-resolved tumor volume of mice changes with different treatments (*n* = 8). (G) tumor weight changes with different treatments (^*^P < 0.05, ^**^P < 0.01, ^***^P < 0.001, ^****^P < 0.0001, *n* = 8). (H) body weight change of mice with different treatments (*n* = 8). (I) H&E stained images of the tumors collected from the mice in all groups (scale bar, 200 μm). CPT, camptothecin; ODCM, orlistat-dopamine conjugates micelle; PBS, phosphate buffered saline.

## Microbial metabolite enhances immunotherapy response by initiating the formation of mature tertiary lymphoid structures

**Rui Zhao^1,2^, Yuxin Chen^1^, Bo Chen^1^, Gang Chen^1^**

^1^The First Affiliated Hospital of Wenzhou Medical University, Wenzhou, Zhejiang Province, China;

^2^Fourth Department of Liver Disease, Beijing Youan Hospital, Capital Medical University, Beijing, China

Address for Correspondence: Gang Chen, Email: chen.gang@wmu.edu.cn

### Abstract

**Background**: Mature tertiary lymphoid structures (mTLS) within tumors exhibit potent anti-tumor effects and may serve as biomarkers to guide treatment selection in cancer patients. However, inducing the formation of mTLS in vivo remains a key challenge in the field of tumor immunology. The gut microbiota has also been implicated in the regulation of tumor immunity. Our previous work identified an association between the genus Lachnoclostridium and the presence of mTLS in hepatocellular carcinoma (HCC). **Methods**: To investigate the relationship between the two, we first administered an antibiotic cocktail to C57/BL6 mice to deplete the gut microbiota. A primary HCC model was then established using the Sleeping Beauty transposon system (cMyc–nRAS-SB), followed by gavage with *Lachnoclostridium massiliosenegalense* (*L.m*). Fecal samples were collected for 16S rRNA sequencing to evaluate microbial composition and confirm bacterial colonization. Subsequently, combined αPD-1 treatment was administered on days 8, 11, and 14. Mice were sacrificed at week 14, and serum and liver tissue samples were collected for tumor burden assessment, transcriptomic sequencing, and metabolomic analysis to identify differential metabolites. Metabolite-based therapeutic experiments were conducted, along with flow cytometry to evaluate treatment efficacy. After confirming that *L.m* synergizes with αPD-1 to enhance anti-tumor immunity, we further explored the mechanism by which microbial metabolites promote the accumulation of tumor-infiltrating B lymphocytes, thereby inducing the formation of mTLS. **Results**: *L.m* treatment significantly reduced tumor burden in mice. Histopathological and transcriptomic analyses of liver tissues revealed that microbial supplementation enhanced the efficacy of αPD-1 and successfully induced the formation of intratumoral mTLS, accompanied by an elevated TLS score. Notably, administration of the single bacterial strain alone was sufficient to induce TLS formation. Metabolomic profiling of serum and liver tissues showed significant enrichment of ursodeoxycholic acid (UDCA), a bacterial metabolite, in both peripheral blood and liver tissues. UDCA administration reduced tumor burden and enhanced the effect of αPD-1. Flow cytometry confirmed a significant increase in tumor-infiltrating lymphocytes, particularly CD19^+^ B cells, after bacterial treatment. Further evidence indicated that microbial metabolites promote the formation of mTLS. **Conclusion**: Our findings demonstrate for the first time that gut microbiota can enhance the efficacy of αPD-1 in HCC by inducing mTLS formation and augmenting anti-tumor immunity. The metabolite UDCA contributes to this process by promoting B lymphocyte infiltration into tumors. Given that UDCA is already a clinically approved drug, our results highlight the considerable translational potential of microbiota-based interventions and drug repurposing strategies.

**How to cite this abstract**: Zhao R, Chen Y, Chen B, Chen G. Microbial metabolite enhances immunotherapy response by initiating the formation of mature tertiary lymphoid structures. J Transl Intern Med 2025; 13: A628-A629.

**Figure:**
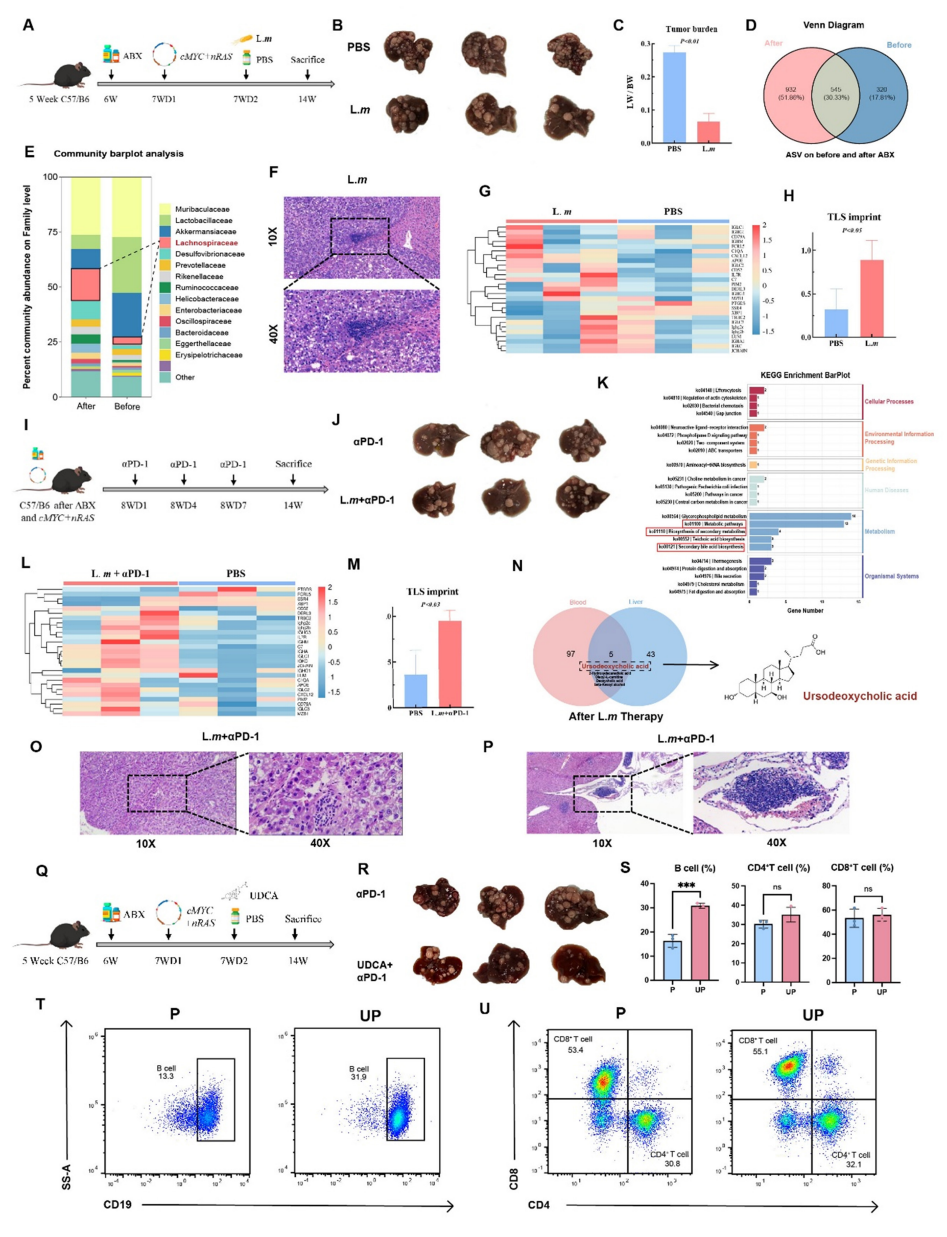
(A) Schematic of the establishment of a primary HCC model in C57/BL6 mice. (B) Macroscopic view of tumors post-*L.m* treatment. (C) Tumor burden following *L.m* treatment. (D) Venn diagram depicting distinct microbiota composition between pre- and post-treatment groups. (E) Differential microbiota enrichment analysis identifies Lachnospiraceae as the most significantly enriched taxon. (F) Representative histology post-*L.m* treatment, showing an immature tertiary lymphoid structure. (G, H) Bulk RNA-seq of liver tissue post- *L.m* treatment shows an elevated TLS signature score. (I) Schematic of the αPD-1 treatment regimen. (J) Macroscopic view of tumors post-combination therapy with *L.m* and αPD-1, indicating an enhanced immunotherapy response. (K) Untargeted metabolomics post- *L.m* treatment reveals enrichment of the secondary bile acid biosynthesis pathway. (L, M) Bulk RNA-seq of liver tissue post-combination therapy with *L.m* and αPD-1 shows a further increased TLS signature score. (N) UDCA as a key metabolite significantly elevated in both liver and blood post-*L.m* treatment. (O, P) Representative histology post-combination therapy with αPD-1 or *L.m* and αPD-1, showing (O) an immature and (P) a mature tertiary lymphoid structure. (Q) Schematic of the UDCA treatment regimen. (R) Macroscopic view of tumors post-combination therapy with UDCA and αPD-1. (S) Quantification of liver-infiltrating immune cells by flow cytometry post-combination therapy with UDCA and αPD-1. (T, U) Representative flow cytometry plots post-combination therapy with UDCA and αPD-1.

## Long-term nucleos(t)ide analog treatment promotes continuous decline of HBsAg in patients with chronic hepatitis B

**Yifan Guo^1,2#^, Yan Liu^3^, Dong Ji^1,2^**

^1^Senior Department of Hepatology, the Fifth Medical Center of PLA General Hospital, Beijing, China;

^2^Peking University 302 Clinical Medical School, Beijing, China;

^3^Senior Department of Infectious Diseases, the Fifth Medical Center of PLA General Hospital, Beijing, China

Address for Correspondence: Dong Ji, Email: jidg302@126.com

### Abstract

**Background and Objectives**: The current ideal goal for the treatment of chronic hepatitis B (CHB) is to achieve clinical cure, which can significantly reduce the risk of liver cirrhosis and hepatocellular carcinoma (HCC) as well as improve long-term outcomes. For some patients, the addition of peginterferon α-2b (PEG IFN α-2b) in treatment has been proven to effectively achieve clinical cure and is suitable for young patients with good baseline conditions and a pursuit of a limited course of treatment. However, its clinical application is limited by adverse effects: uncertain efficacy and stringent patient selection criteria. Some studies indicate that long-term nucleos(t)ide analog (NAs) monotherapy with good adherence significantly reduces liver fibrosis markers in CHB patients, demonstrating its potential to reverse fibrosis and promote clinical cure. This study aims to identify baseline factors predictive of HBsAg decline for CHB patients under long-term NAs treatment. **Methods**: A total of 513 patients with CHB who received NAs at the Fifth Medical Center of the PLA General Hospital from January 2012 to June 2025 without PEG IFN α-2b treatment were included in the study. The clinical data were retrospectively collected, including the basic information of the patients, the course of antiviral treatment (defined as the time from the start of antiviral drug use to the most recent hospital visit), and the test and examination indicators when antiviral treatment was initiated. The endpoint was defined as HBsAg level less than 250 IU/mL for the first time. Multivariate logistic regression was used to analyze the independent risk factors, and trend analysis of subgroups was conducted for the independent risk factors. **Results**: Among the 513 enrolled patients, 357 were male (69.6%), 186 patients (36.3%) achieved significant HBsAg decline. Multivariate logistic regression analysis revealed that antiviral therapy duration of 5-10 years (OR = 3.005, 95% CI: 1.519-5.944), < 10 years (OR = 8.837, 95% CI: 4.403-17.737, *P* < 0.001), baseline HBsAg ≤ 2000 IU/mL (OR = 0.403, 95% CI: 0.251-0.649, *P* < 0.001), HBeAg-negative status (OR = 5.471, 95% CI: 3.050-9.815, *P* < 0.001), and PLT < 100 × 109/L (OR = 1.918, 95% CI: 1.065-3.452, *P* = 0.030) were significantly associated with HBsAg reduction, whereas age at treatment initiation, NAs drug type, and baseline HBV DNA or ALT levels showed no correlation. Subgroup analyses demonstrated a duration-dependent effect, with longer treatment correlating with greater HBsAg decline. Patients with baseline HBsAg ≤ 2000 IU/mL achieved significantly lower HBsAg levels at follow-up compared to those with < 2000 IU/mL (*P* < 0.05), while platelet count and HBeAg status also significantly influenced the HBsAg levels of patients. **Conclusion**: This study identifies key baseline factors: regardless of age or antiviral drug type, HBeAg-negative patients with lower baseline HBsAg levels and PLT < 100 × 109/L demonstrated higher HBsAg clearance rates. This phenomenon may be attributed to the long half-life of cccDNA, which requires sustained viral suppression over time to ultimately achieve HBsAg clearance. The duration-dependent therapeutic effect and stratified response by baseline HBsAg levels provide clinically actionable insights for optimizing treatment strategies toward functional cure.

**How to cite this abstract**: Guo Y, Liu Y, Ji D. Long-term nucleos(t)ide analog treatment promotes continuous decline of HBsAg in patients with chronic hepatitis B. J Transl Intern Med 2025; 13: A630-A631.

**Figure 1:**
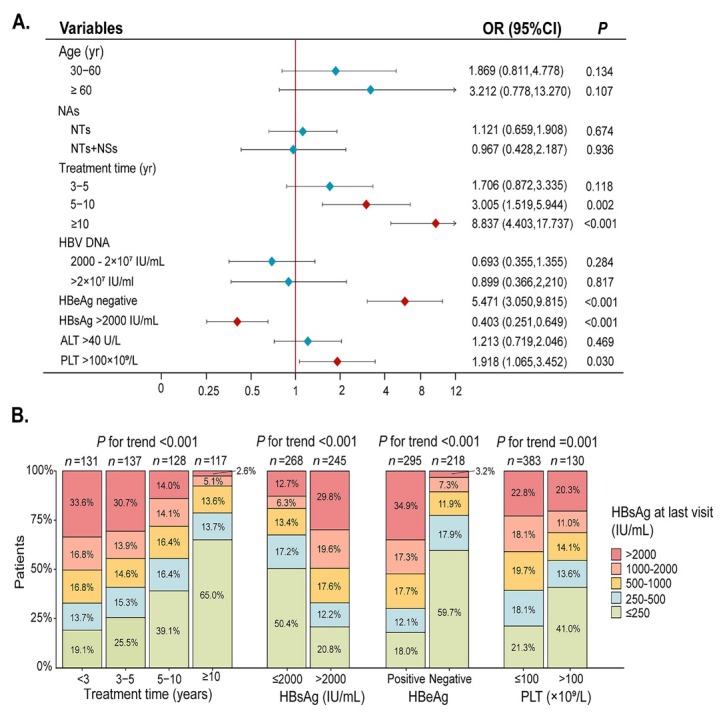
(A) Multivariable logistic regression analysis of the factors associated with HBsAg reduction. (B) Trend test for the clinical characteristics of the patients stratified by HBsAg. NAs, nucleos(t)ide analogues; NTs, nucleotide analogues (including TDF, ADV, TAF); NSs, nucleoside analogues (including ETV, LAM); HBsAg, hepatitis B surface antigen; HBeAg, hepatitis Be antigen; PLT, platelet.

## Risk stratification and mortality of acute-on-chronic liver failure across different diagnostic criteria: A multicenter cohort study

**Wenling Wang^1,2#^, Manman Xu^1,2#^, Yu Wu^1,2^, Yanrong Yang^1,2^, Xiao Che^1,2^, Jianhui Lin^3^, Junfeng Li^4^, Hongchao Chen^5^, Xiaogang Xiang^6^, Jia Yao^7^, Shaoli You^8^, Yingli He^9^, Rongqi Wang^10^, Yan Huang^11^, Yu Chen^1,2^**

^1^Fourth Department of Liver Disease (Difficult & Complicated Liver Diseases and Artificial Liver Center), Beijing Youan Hospital, Capital Medical University, Beijing, China;

^2^Beijing Key Laboratory of Liver Regeneration and Artificial Liver Transformation Research, Beijing, China;

^3^Department of Liver Diseases, Mengchao Hepatobiliary Hospital of Fujian Medical University, Fuzhou, Fujian Province, China;

^4^Infectious Disease Research Laboratory, The First Hospital of Lanzhou University, Lanzhou, Gansu Province, China;

^5^Gastroenterology Department, Western Theater Command General Hospital of PLA, Chengdu, Sichuan Province, China;

^6^Department of Infectious Diseases, Ruijin Hospital, Shanghai Jiao Tong University School of Medicine, Shanghai, China;

^7^Third Hospital of Shanxi Medical University, Shanxi Bethune Hospital, Shanxi Academy of Medical Sciences, Tongji Shanxi Hospital, Taiyuan, Shanxi Province, China;

^8^Hepatology Department, The Fifth Medical Center of Chinese PLA General Hospital, Beijing, China;

^9^Clinical Research Center for Infectious Diseases, The First Affiliated Hospital of Xi'an Jiaotong University, Xi'an, Shaanxi Province, China;

^10^Department of Traditional and Western Medical Hepatology, The Third Hospital of Hebei Medical University, Shijiazhuang, Hebei Province, China;

^11^Department of Infectious Diseases, Hunan Key Laboratory of Viral Hepatitis, Xiangya Hospital, Central South University, Changsha, Hunan Province, China

^#^These authors contributed equally to this work.

Address for Correspondence: Yu Chen, Email: chybeyond1071@ccmu.edu.cn

### Abstract

**Background and Objectives**: Acute-on-chronic liver failure (ACLF) is a heterogeneous syndrome with high short-term mortality and a substantial global health burden. Despite multiple proposed definitions—including those from APASL, COSSH, and EASL—universally accepted and standardized diagnostic criteria are lacking, resulting in inconsistent patient stratification and outcome prediction. The Chinese Medical Association (CMA) has released the new China Guidelines for the Diagnosis and Treatment of ACLF (2025), and this study aims to evaluate the compatibility and comprehensiveness of these guidelines. **Methods**: In this prospective, multicenter study, clinical data were collected between September 2023 and May 2025 across multiple centers in China within the CGSLD consortium. Eligible patients were those with acute decompensation of cirrhosis or severe liver injury who met predefined thresholds (total bilirubin [TB] ≥ 5 mg/dL and international normalized ratio [INR] ≥ 1.5). Baseline characteristics, clinical course, and outcomes were analyzed to compare existing diagnostic frameworks (APASL-, COSSH-, EASL- and CMA-ACLF). **Results**: Out of 1138 patients initially screened, 1038 with acute deterioration of chronic liver disease of various etiologies were enrolled in this prospective, multicenter study. All patients (100%) fulfilled the APASL-ACLF (2025) criteria, with 28- and 90-day liver transplant (LT)-free mortality rates of 22.9% and 35.5%, respectively. Among them, 83.1% (863) and 35.5% (368) also met the COSSH-ACLF and EASL-ACLF criteria, with corresponding 28-/90-day LT-free mortality rates of 26.8%/41.4% and 36.3%/53.7%, respectively. According to the COSSH criteria, 55.7% (481) of patients were classified as grade 1, 35.8% (309) as grade 2, and 8.5% (73) as grade 3. Based on the EASL criteria, 30.7% (113) were classified as grade 1, 55.4% (204) as grade 2, and 13.9% (51) as grade 3. In COSSH-ACLF grade 1, the presence of acute kidney injury (AKI) or extrahepatic organ failure was associated with significantly higher 28-/90-day LT-free mortality compared with those without such complications (31.8%/36.4% *vs*. 14.1%/25.7%; *P* < 0.05). Among patients with ACLF complicated by AKI or extrahepatic organ failure, 3.4% (6/174) had total bilirubin (TB) levels between 5.0 and 7.5 mg/dL, 13.7% (24/174) between 7.5 and 12.0 mg/dL, and 82.7% (144/174) above 12.0 mg/dL. Their corresponding 28-/90-day LT-free mortality rates were 50.0%/50.0%, 35.0%/50.0%, and 55.3%/66.7%, respectively, indicating that even patients with TB < 5.0 mg/dL exhibited high mortality. To harmonize the different diagnostic definitions, this study, in accordance with the newly released CMA guidelines, reclassified ACLF patients into CGSLD-ACLF I and CGSLD-ACLF II. CGSLD-ACLF I, defined as acute severe liver injury on the background of chronic liver disease characterized by TB ≥ 12 mg/dL together with INR ≥1.5, was identified in 67.7% (702) of patients, with corresponding 28- and 90-day LT-free mortality rates of 20.7% and 35.2%, respectively. Stratification according to INR (1.5 ≤ INR < 2.0, 2.0 ≤ INR < 2.5, and INR ≥ 2.5) identified early, intermediate, and advanced stages, with corresponding 28-/90-day LT-free mortality rates of 8.1%/19.7%, 22.1%/33.7%, and 40.0%/58.2%, respectively. CGSLD-ACLF II was defined as a marked elevation in TB and INR ≥1.5, accompanied by the development of renal dysfunction (creatinine 1.5–2.0 mg/dL) or extrahepatic organ failure (renal, cerebral, respiratory, or circulatory) within one week. The critical threshold for TB has not been definitively established, but this study indicates that a TB level above 5 mg/dL is associated with a mortality rate exceeding 15%. We recommend expanding the sample size in future studies to further validate this threshold. This criterion was met by 16.8% (174) of patients, with 28- and 90-day LT-free mortality rates of 49.3% and 63.6%, respectively. Patients were further stratified into early, intermediate, and advanced stages according to the presence of renal dysfunction, single extrahepatic organ failure, or multiple extrahepatic organ failures. The corresponding 28-/90-day LT-free mortality rates were 25.7%/37.1%, 58.0%/66.7%, and 70.8%/91.7%, respectively. The staging of CGSLD-ACLF I reflects the progressive continuum from chronic disease to increasing severity, highlighting the dynamic process of disease aggravation. In contrast, the staging of CGSLD-ACLF II primarily indicates the severity of the condition, thereby facilitating risk stratification and management. A total of 15.6% (162) of patients did not meet the criteria for either CGSLD-ACLF I or II, and their 28- and 90-day LT-free mortality rates were 5.5% and 8.6%, respectively. **Conclusion**: The CGSLD-ACLF classification provides a refined framework for assessing disease severity. This approach enhances risk stratification and facilitates timely, intensive interventions to improve patient outcomes. Moreover, it increases the diagnostic sensitivity and specificity for ACLF.

**How to cite this abstract**: Wang W, Xu M, Wu Y, Yang Y, Che X, Lin J, *et al*. Risk stratification and mortality of acute-on-chronic liver failure across different diagnostic criteria: A multicenter cohort study B. J Transl Intern Med 2025; 13: A632-A633.

**Figure:**
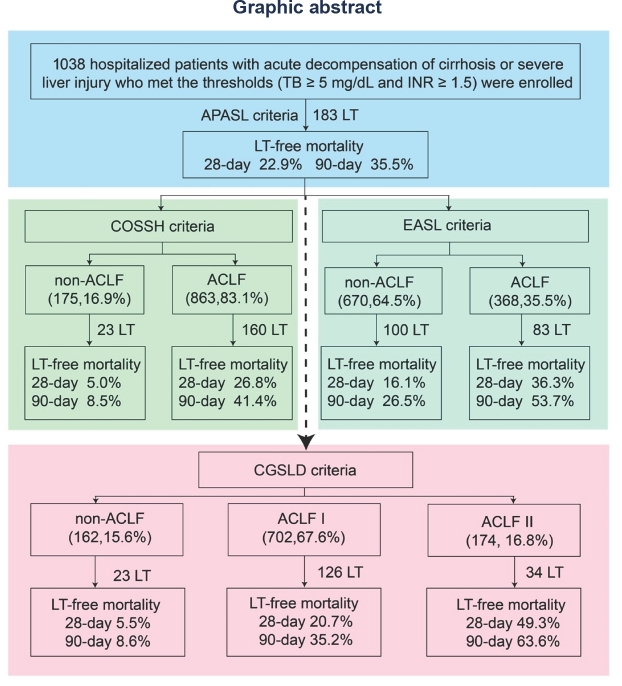
ACLF, acute-on-chronic liver failure; TB, total bilirubin; INR, international normalized ratio; LT, liver transplantation; APASL, Asian Pacific Association for the Study of the liver; COSSH, Chinese Group on the Study of Severe Hepatitis B; EASL, European Association for the Study of Liver-Chronic Liver Failure; CGSLD, Chinese Group for the Study of Severe Liver Diseases.

## Novel compound heterozygous *AGL* variants (c.3836G<A/c.4284T<G) in a chinese child with glycogen storage disease type III

**Pan Liu, Li Tang, Xiaoguai Liu**

Department of infectious diseases, Xi'an Children's Hospital, Xi’an, Shaanxi Province, China

Address for Correspondence: Ruibing Li, Email: liruibing@plagh.org; Mianyang Li, Email: limianyang@301hospital.com.cn

### Abstract

**Introduction**: Glycogen storage disease type III (GSD III) is an autosomal recessive disorder caused by variants in *AGL* gene. Diagnosis relies on combined clinical assessment and genetic variant analysis. We report a GSDIII pedigree characterized by persistent transaminitis and hepatomegaly, analyzing its clinical and molecular genetic features to enhance clinicians' recognition of this disease and broaden the mutational spectrum of *AGL*. **Case description**: A 4-year-4-month-old boy presenting with abnormal liver function over 2 years was referred to our hospital. Subsequent intermittent monitoring revealed persistent transaminitis with ALT 132–263 U/L and AST 86–199 U/L. Growth retardation was observed by parents since 1 year of age, with current anthropometric measurements showing weight 13.5 kg (weight-for-age Z-score: -1.83) and height 95.5 cm (height-for-age Z-score: -2.35), while psychomotor development remained normal. Physical examination revealed abdominal distension and severe hepatomegaly. Abdominal ultrasound demonstrated hepatomegaly with a slightly hyperechoic area (7.5 × 5.6 mm) in the right hepatic lobe suggestive of hemangioma. Liver biopsy was performed, showing diffusely swollen hepatocytes with rarefied, pale cytoplasm resembling plant cells, glycogenated nuclei, and compressed hepatic sinusoids. Whole exome sequencing of the proband and his parents identified compound heterozygous variants in the *AGL* gene (NM_000642.3): a novel c.3836G<A (p.Arg1279Lys) in exon 28 and a novel c.4284T<G (p.Tyr1428*) in exon 32, both unreported in existing databases. Sanger confirmation showed maternal/paternal inheritance of these novel variants. The missense variant c.3836G<A (p.Arg1279Lys) substitutes arginine with lysine at residue 1279 of the AGL protein. Its population frequency in Exomes databases is 0.000017 (PM2). Comparative analysis of AGL orthologs across eight eukaryotic species (Homo sapiens, Pan troglodytes, Macaca mulatta, Bos taurus, Canis lupus, Equus caballus, Mus musculus, and Rattus norvegicus) revealed evolutionary conservation of R1279. Homology modeling of wild-type AGL structure *via* SWISS-Model and PyMol visualization demonstrated that the R1279K substitution disrupts hydrogen bonding networks and alters bond lengths, thereby perturbing protein conformation and function. Pathogenicity predictions by SIFT4G, MutationAssessor, and REVEL support deleterious effects (PP3). The variant was detected in trans with a pathogenic variant (PM3), and the patient’s phenotype highly matches glycogen storage disease (PP4). Per ACMG guidelines, this variant is classified as likely pathogenic (PM2+PM3+PP3+PP4). The nonsense variant c.4284T<G (p.Tyr1428*) introduces a premature termination codon, resulting in a loss-of-function allele (PVS1). It is absent from gnomAD population controls (PM2) and was identified in trans with a pathogenic variant (PM3). According to ACMG criteria, it is classified as pathogenic (PVS1+PM2+PM3). **Conclusion**: We report novel *AGL* variants (c.3836G<A and c.4284T<G) in a Chinese GSD III patient, broadening the mutational spectrum of AGL and highlighting the need for GSD III screening in patients with transaminitis, hepatomegaly, hypoglycemia, and growth retardation.

**How to cite this abstract**: Liu P, Tang L, Liu X. Novel compound heterozygous AGL variants (c.3836G<A/c.4284T<G) in a chinese child with glycogen storage disease. J Transl Intern Med 2025; 13: A634-A635.

**Figure:**
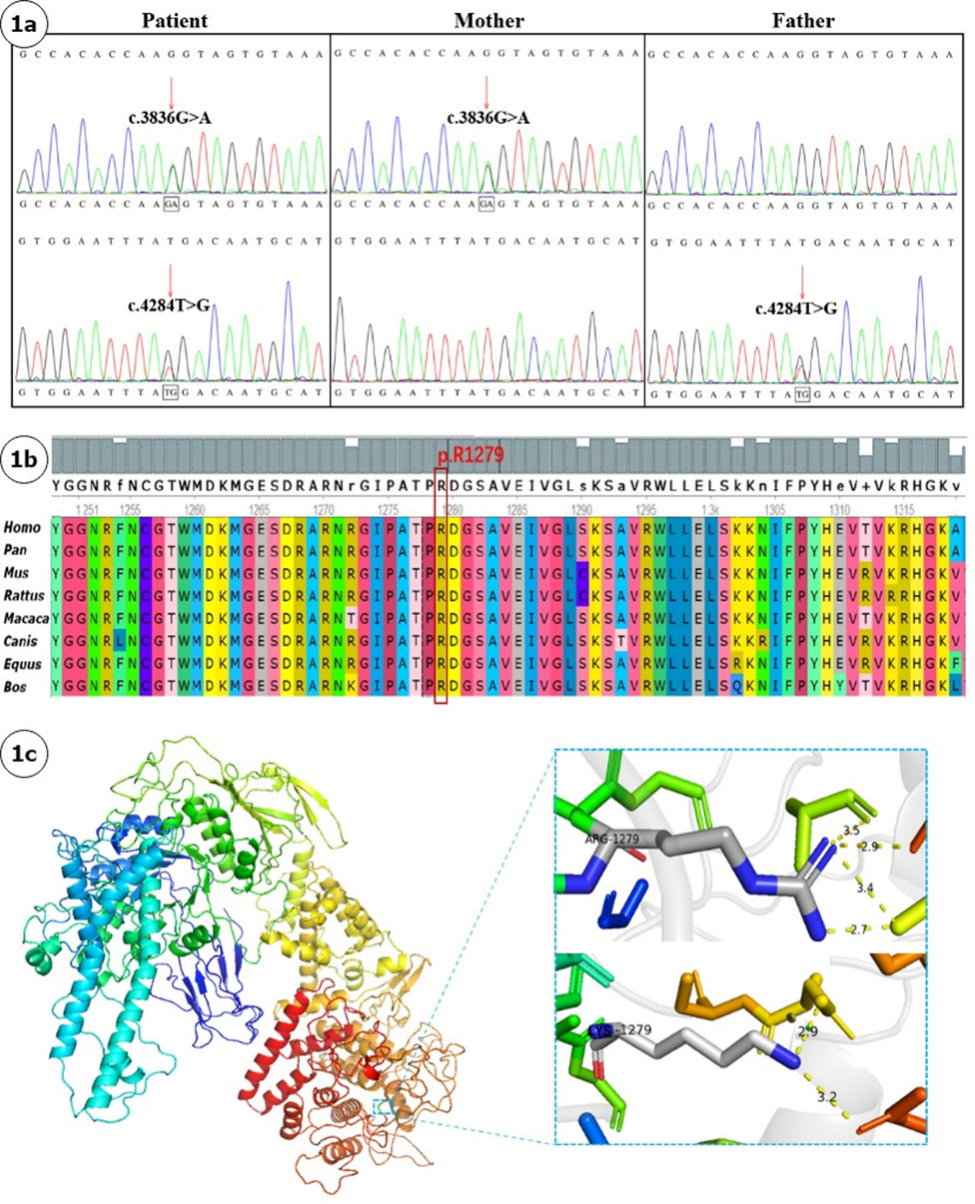
(A). Sanger sequencing chromatograms of AGL variants in the proband and parents Proband showing compound heterozygous variants c.3836G<A and c.4284T<G; Maternal heterozygous variant c.3836G<A; Paternal heterozygous variant c.4284T<G. (B). Cross-species conservation analysis of AGL protein residues Red arrow indicates the evolutionarily conserved residue at position 1279. (C). Structural modeling of wild-type and mutant AGL proteins Wild-type AGL protein with R1279 highlighted (blue box). Upper panel: Wild-type R1279 residue with 5-Å hydrogen-bonding network; Lower panel: Mutant K1279 residue showing hydrogen bond disruption and altered bond lengths.

## Sacral insufficient fracture in an elderly people

**Hao Wang^1^, Kai Yu^1^, Lianhua Li^2^, Jianzheng Zhang^2^**

^1^Department of Orthopedics, Beijing Genertec Aerospace Hospital, Beijing, China;

^2^Department of Orthopedics, Chinese PLA General Hospital, Beijing, China

Address for Correspondence: Jianzheng Zhang, Email: drzhangjianzheng@126.com

### Abstract

**Background**: Sacral insufficiency fracture (SIF)is a well defined fragility fracture but insufficient awareness of SIF currently persists among orthopedic,emergency and geriatric physicians. **Objective**: To heighten clinical characteristics and therapeutic principles of SIF, we presented the diagnostic and treatment process of this case. **Case presentation**: A 73-year old female without trauma history complained severe pain in low back and pubic symphysis for 20 days. She was diagnosed pubic fracture and lumbar spinal stenosis initially The pelvic X-ray showed mild displacement symphysis pubis fracture without dislocation of the joint (Figure 1) and Dual-energy X ray Absorptiometry (DXA) showed T score -3.2 in lumbar and-3.1in left hip. Conservative treatment,which include immobilization, multimodal pain management and osteoporosis management was carried out with limited efficacy. The patient complained sharp pain when turn over in bed. Pelvic CT was performed for further evaluation and it showed sacrum fracture in both alaes and S1 vertebral body (Figure 2). Diagnosis of insufficient sacrum fracturewas supported by CT, DXA and clinical atrauma history. Considering the prolonged pain after conservative treatment and minimal fracture displacement. Minimally invasive surgery (anterior pelvic ring internal fixator for symphysis pubis fracture combined iliosacral screws for sacrum fracture) was implemented. Visual analogue scale decreased from 8 pre-operation to 4 post operation (Figure 3). Osteoporosis management and rehabilitation was carried out postoperation. **Results**: The patient achieved full weight-bearing ambulation with mild pain 4 weeks postoperation and anterior pelvic ring internal fixator was moved six months postoperation (Figure 4). **Conclusion**: 1. Orthopedic, emergency and geriatric physicians should be vigilant against sacral insufficiency fractures in atraumatic elderly patients presenting with low back pain. 2. SIFs could be easily missed on pelvic X-ray because sacrum is poorly visualized and minimal fracture displacementof SIFs. CT and MRI is recommended for diagnosis. 3. The majority of non-displaced SIFs with low level pain could be initially treated conservatively and patients with prolonged pain could benefit significantly from osteosynthesis.

**How to cite this abstract**: Wang H, Yu K, Li L, Zhang J. Sacral insufficient fracture in an elderly people. J Transl Intern Med 2025; 13: A636-A637.

**Figure 1:**
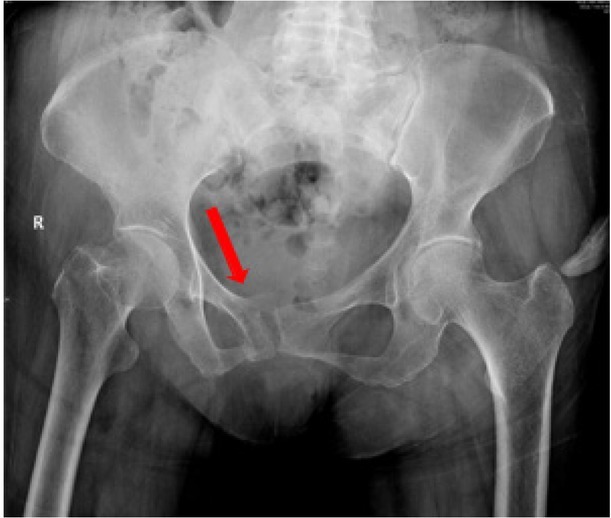
Pelvic X-ray showed mild displacement symphysis pubis fracture without dislocation of the joint.

**Figure 2:**
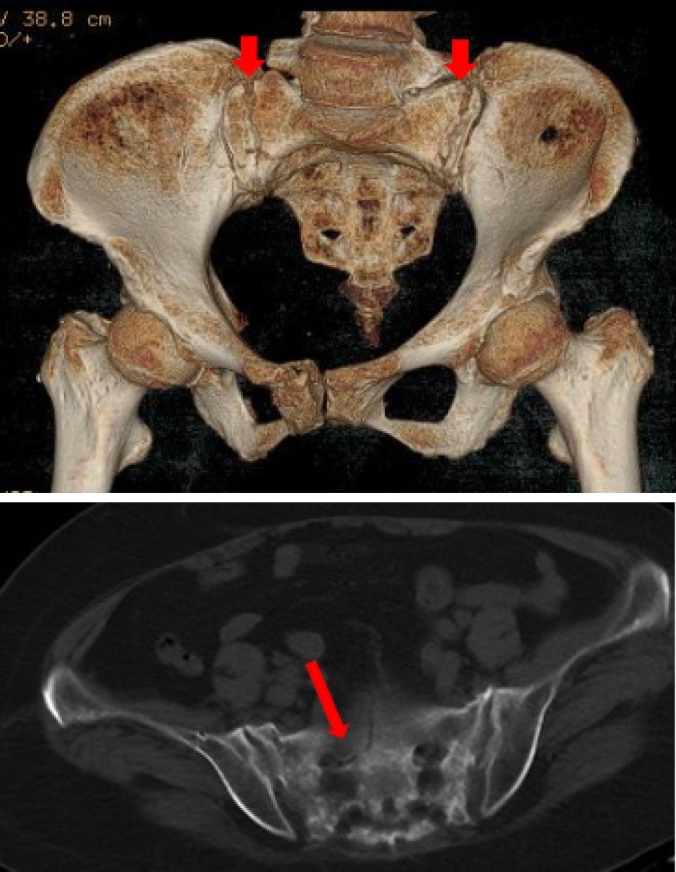
CT examination showed sacrum fracture in both alae of sacrum and vertebral body of S1. CT, computed tomography.

**Figure 3:**
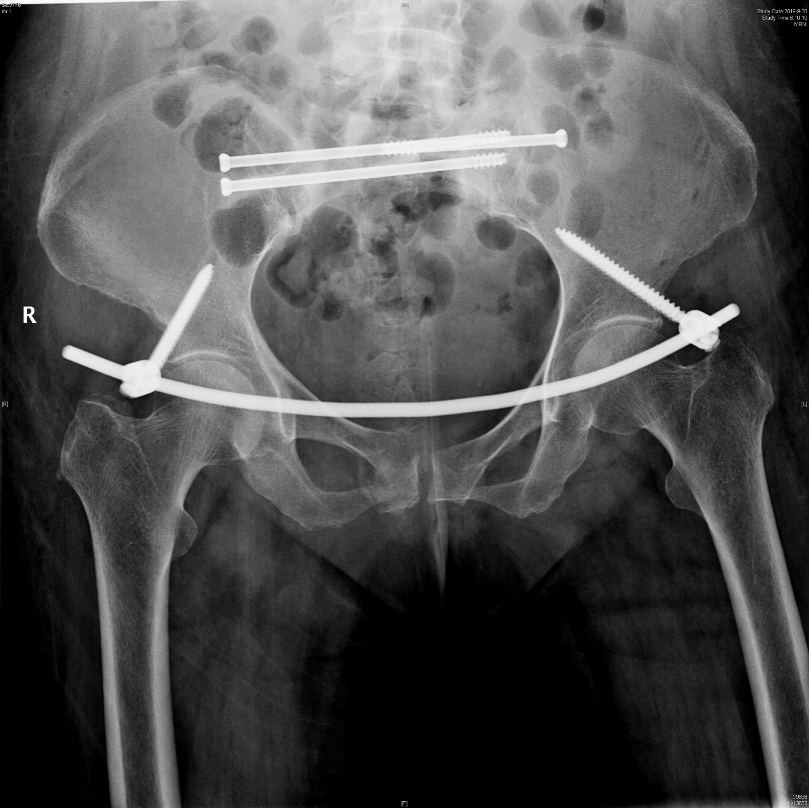
X-ray postoperation showed anterior pelvic ring internal fixator for symphysis pubis fracture combined iliosacral screws for sacrum fracture.

**Figure 4:**
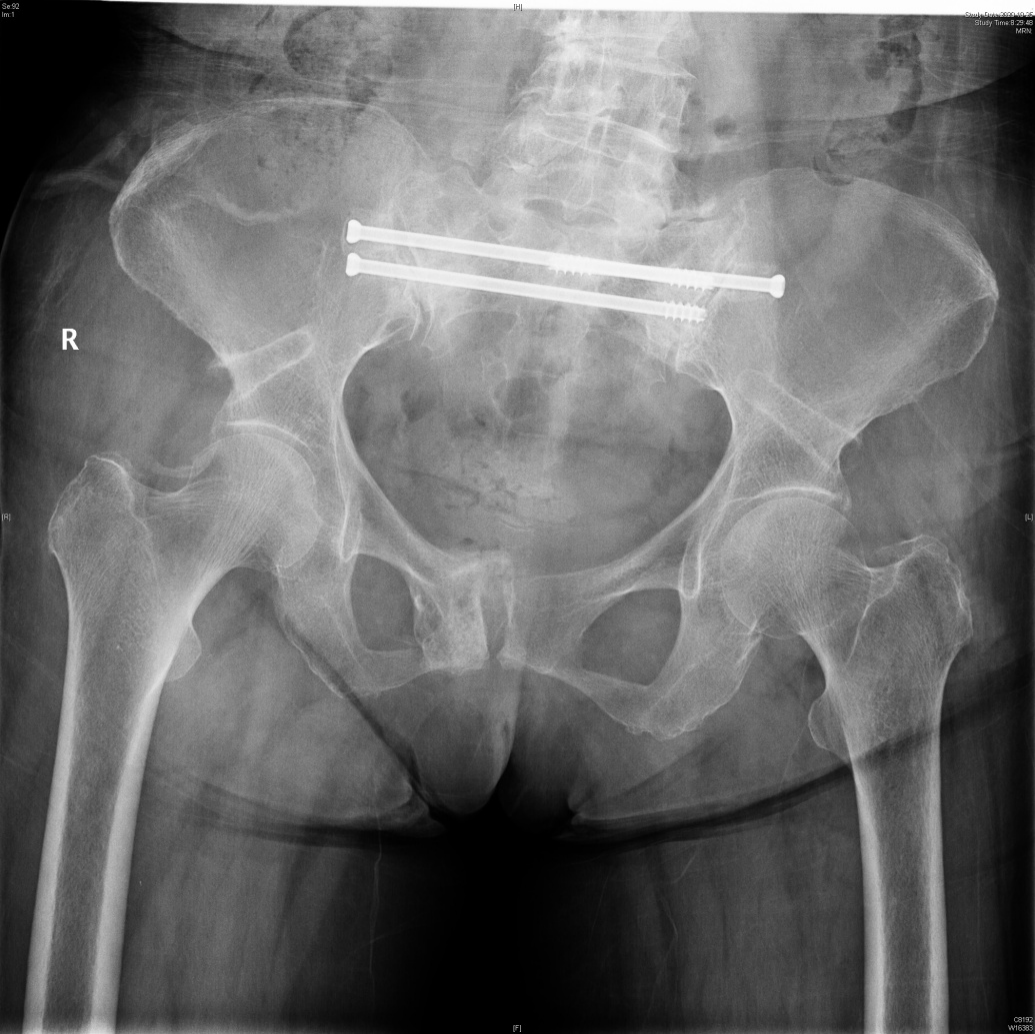
Anterior pelvic ring internal fixator was removed 6 months post-operation and iliosacral screws were reserved.

## Splenectomy reduces post-endotherapy variceal rebleeding in cirrhotic patients: A decade-long multicenter study

**Qiqi Liu^1^, Zhen Zhang^2^, Xinke Wang^3^, Shize Xu^1^, Ping He^3^, Tianru Zhang^3^, Shuhang Wei^1^, Yinuo Yang^1^, Can Zhang^3^, Ping Lv^3^, Yuemin Feng^3^, Guodong Li^2^, Qiang Zhu^4^**

^1^Department of Gastroenterology, Shandong Provincial Hospital, Cheeloo College of Medicine, Shandong University, Jinan, Shandong Province, China;

^2^Department of Gastroenterology, The First Affiliated Hospital of Shandong First Medical University & Shandong Provincial Qianfoshan Hospital, Jinan, Shandong Province, China;

^3^Department of Gastroenterology, Shandong Provincial Hospital Affiliated to Shandong First Medical University, Jinan, Shandong Province, China;

^4^Department of Infectious Disease, Shandong Provincial Hospital Affiliated to Shandong First Medical University, Jinan, Shandong Province, China

Address for Correspondence: Qiang Zhu, Email: zhuqiang@sdu.edu.cn

### Abstract

**Background**: This study aims to evaluate the efficacy of splenectomy in preventing rebleeding among cirrhotic patients with recurrent variceal bleeding, and to establish a tailored prediction model to facilitate early identification of high-risk rebleeding candidates. **Methods**: Eligible patients hospitalized for endoscopic treatment due to esophagogastric variceal bleeding from January 2010 to January 2024 were enrolled in the study, and then they underwent a 12-month follow-up for detection of recurrent bleeding. Univariable and multivariable logistic regression analyses were applied to detect the independent protective effect of splenectomy. LASSO regression and extreme gradient boosting (XGBoost) model, with Shapley additive explanations (SHAP) were processed to construct a rebleeding predictive nomogram model. The optimal threshold was determined by maximizing the Youden index derived from receiver operating characteristic (ROC) curve analysis. **Results**: Logistic regression analyses showed that there were five variables closely associated with the occurrence of rebleeding, including splenectomy, last bleeding interval time, portal vein diameter, “endoscopic injection sclerotherapy (EIS) plus endoscopic cyanoacrylate injection (ECI)” of treatment methods, and albumin (ALB). Interaction analysis showed that the protective effect of splenectomy remained significant within each subgroup, and no interaction existed. Additionally, ALB and high density lipoprotein cholesterol (HDL-C) demonstrated statistically significant mediating effects in the association between splenectomy. Then, the rebleeding predictive model (SDPAH points) was developed and predictive efficiency was evaluated in the training cohort, internal validation cohort, and external validation cohort, with the AUC of 0.763, 0.707, and 0.819, respectively. The optimal cut-off value of SDPAH points was determined to be 167.1 points, demonstrating satisfactory discriminative performance. There was a U-shaped association between portal vein diameter and rebleeding in one year, with an optimal threshold of 1.46 cm. **Conclusion**: Prior treatment with splenectomy independently reduces 1-year rebleeding post-endotherapy in recurrent bleeding cirrhosis patients. Our rebleeding predictive model and threshold stratify high-risk patients, offering liver transplant-ineligible cases a viable alternative.

**How to cite this abstract**: Liu Q, Zhang Z, Wang X, Xu S, He P, Zhang T, *et al*. Splenectomy reduces post-endotherapy variceal rebleeding in cirrhotic patients: A decade-long multicenter study. J Transl Intern Med 2025; 13: A638.

## Unveiling a plasma metabolomic signature for predicting glucocorticoid-induced recompensation in autoimmune hepatitis-associated decompensated cirrhosis

**Xiaolong Lu^1^, Zhenwei Qian^1^, Long Rui^2^, Mian Zhou^2^, Huan Xie^1^, Lin Han^1^, Ying Sun^1^**

^1^Department of Hepatology, the Fifth Medical Center of PLA General Hospital, Beijing, China;

^2^Division of Gastroenterology, the Hospital of 82nd Group Army PLA, Baoding, Hebei Province, China

Address for Correspondence: Ying Sun, Email: sunying_302@163.com

### Abstract

**Background**: Patients with autoimmune hepatitis (AIH)-associated decompensated cirrhosis face a poor prognosis, and glucocorticoid therapy is commonly employed in their management. However, the use of corticosteroids in decompensated patients remains controversial due to the potential risk of serious complications such as infections, coupled with the inconsistent treatment response observed among individuals. The absence of reliable biomarkers to predict therapeutic outcomes poses a significant challenge in clinical decision-making. Metabolomics, which enables systematic characterization of small-molecule metabolite alterations in disease states, offers a promising approach to identify predictive biomarkers of treatment response and to elucidate underlying mechanisms. **Methods**: This study is based on a prospective clinical cohort of AIH patients. Untargeted metabolomic analysis was performed on baseline plasma samples from 42 AIH patients with decompensated cirrhosis who received glucocorticoid therapy, using liquid chromatography coupled with high-resolution mass spectrometry (LC-HRMS). Based on treatment outcomes, patients were divided into a recompensation (RC) group (*n* = 28) and a sustained decompensated (DC) group (*n* = 14). Statistical analysis was conducted using the MetaboAnalyst platform, including parametric/non-parametric tests, fold-change analysis, principal component analysis (PCA), and orthogonal partial least squares-discriminant analysis (OPLS-DA) to screen for differentially abundant metabolites between the groups. Further Kyoto Encyclopedia of Genes and Genomes (KEGG) pathway enrichment analysis was performed on the differential metabolites to identify key metabolic pathways associated with treatment response. **Results**: Untargeted metabolomic profiling identified 2234 metabolites in positive ion mode and 944 in negative ion mode. PCA indicated a separation trend between the two groups (cumulative explained variance: 25.8%), and the OPLS-DA score plot further confirmed significant differences in metabolic profiles (Figure 1). Differential metabolites were selected based on the following criteria: (1) fold change ≥ 2 or ≤ 0.5; (2) FDR < 0.05; (3) variable importance in projection (VIP) ≥ 1. In total, 36 metabolites were significantly up-regulated and 77 were down-regulated in the recompensation group (volcano plot and heatmap in Figure 2; Table 1 shows the top 30 differential metabolites). KEGG pathway enrichment analysis revealed that these differential metabolites were significantly enriched in glycerophospholipid metabolism, primary bile acid biosynthesis, inositol phosphate metabolism, glycosylphosphatidylinositol (GPI)-anchor biosynthesis, and linoleic acid metabolism (Figure 3). **Conclusion**: This study demonstrates that AIH-associated decompensated cirrhosis patients with high baseline levels of specific plasma metabolites are more likely to achieve recompensation after glucocorticoid therapy. These differential metabolites are primarily involved in key pathways such as glycerophospholipid metabolism and primary bile acid biosynthesis, providing novel metabolic markers for prognostic evaluation and a theoretical foundation for developing individualized treatment strategies.

**How to cite this abstract**: Lu X, Qian Z, Rui L, Zhou M, Xie H, Han L, *et al*. Unveiling a plasma metabolomic signature for predicting glucocorticoid-induced recompensation in autoimmune hepatitis-associated decompensated cirrhosis. J Transl Intern Med 2025; 13: A639-A642.

**Figure 1:**
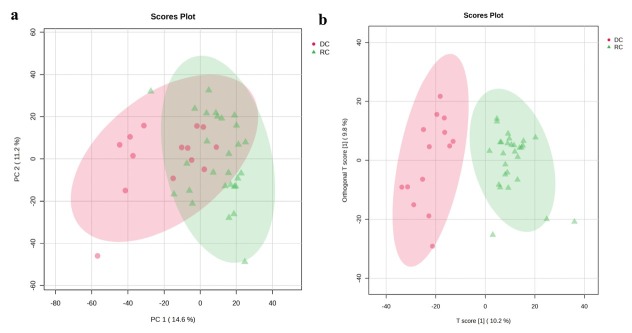
Multivariate model evaluation and validation. a. PCA score plot. b. OPLS-DA score plot.

**Figure 2:**
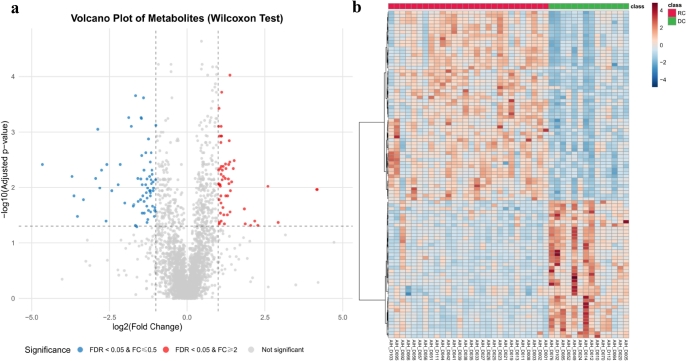
Differential metabolite analysis. a. Volcano plot of differential metabolites. b. Heatmap of differential metabolites.

**Figure 3:**
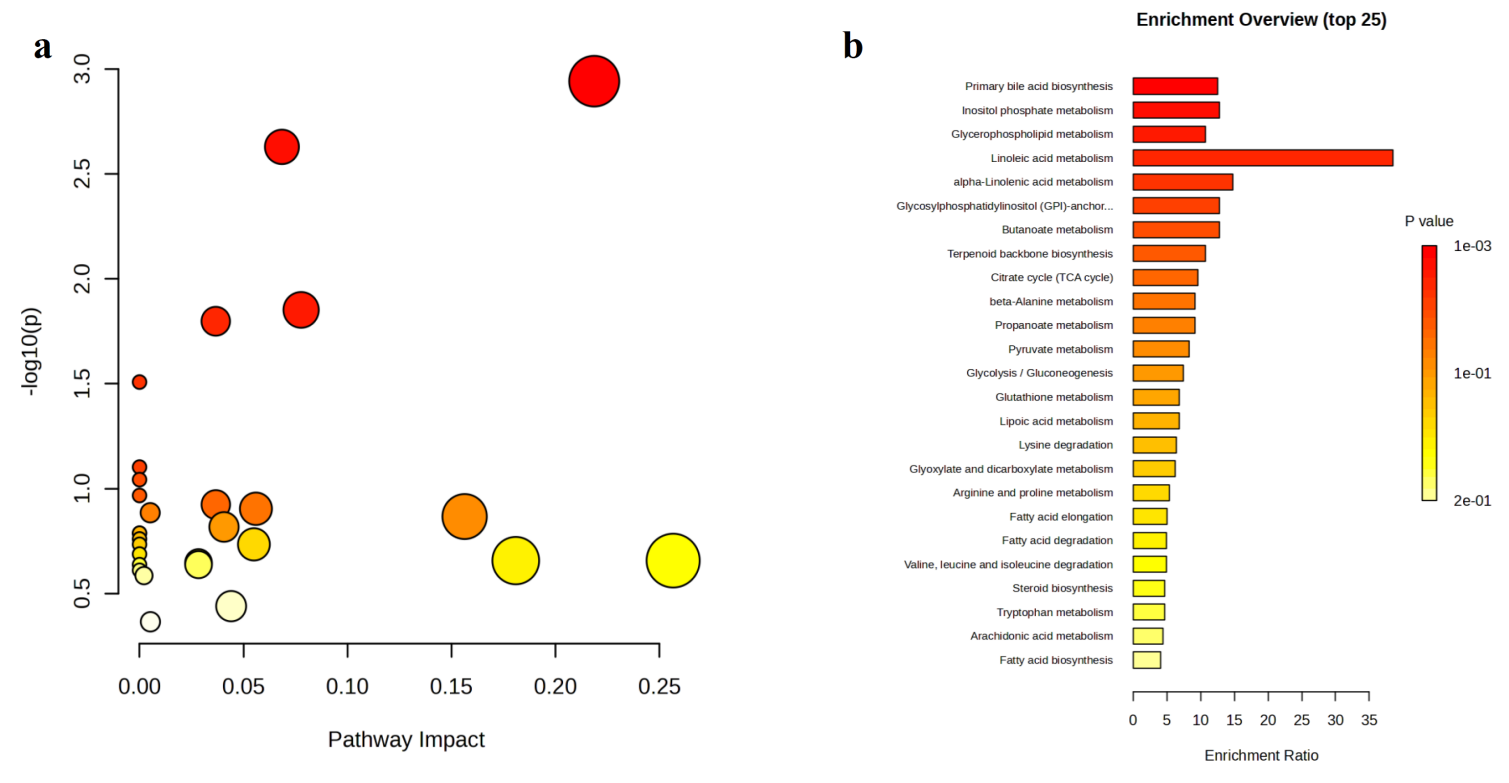
KEGG Pathway Enrichment Analysis. a. Bubble plot. b. Bar chart.

**Table 1:** Top 30 differential metabolites between recompensation and sustained decompensation groups

Metabolite Name	Log2 (FC)	VIP	FDR	Regulation
Phosphatidylcholine (18:0/14:0)	-1.165	2.40	0.001	down
Diacylglycerol (16:0/18:1 (9Z))	-1.233	2.37	0.000	down
1-Lysophosphatidylethanolamine (20:0)	1.095	2.36	0.005	up
Sphingomyelin (d18:2/10:0)	1.073	2.34	0.000	up
Phosphatidylethanolamine (18:1/18:2)	-1.850	2.30	0.000	down
Phosphatidylcholine (16:0/16:0)	-1.005	2.27	0.001	down
Sphingomyelin (d18:0/11:0)	1.072	2.22	0.000	up
Phosphatidylcholine (18:1 (11Z)/14:0)	-1.438	2.19	0.002	down
5-Keto-D-gluconate	-2.564	2.18	0.018	down
Glutamyltyrosine	-1.147	2.15	0.007	down
Prolylphenylalanine	1.138	2.15	0.007	up
Sphingomyelin (d16:1/18:1 (12Z)-2OH (9,10))	-1.258	2.14	0.003	down
Glycocholic acid	-2.128	2.14	0.001	down
Alkylacylglycerol (O-16:0/18:1/18:2)	-1.772	2.13	0.007	down
Galacturonate	-2.746	2.12	0.014	down
Myristoleic acid	-1.817	2.11	0.001	down
Palmitoleic acid	-1.794	2.10	0.001	down
Phosphatidylcholine (16:0/14:0)	-1.107	2.10	0.005	down
Diacylglycerol (29:4)	-1.303	2.10	0.005	down
Phytoglycolipid	1.007	2.09	0.001	up
Oxooctadecadienoic acid	-1.412	2.09	0.001	down
Palmitoleoylcarnitine	-1.345	2.06	0.002	down
Sphingomyelin (d18:1/21:0)	1.039	2.06	0.000	up
Ceramide (d18:1/15:0+hO)	1.031	2.05	0.005	up
(+)-Lysergic acid	1.448	2.05	0.003	up
L-cis-3-Amino-2-pyrrolidinecarboxylic acid	-1.282	2.04	0.022	down
trans-Hexadec-2-enoylcarnitine	-1.619	2.03	0.007	down
Ceramide (d18:1/23:0)	1.003	2.03	0.004	up
Cardiolipin (22:2_20:5)	1.023	2.03	0.006	up
3-Epireserpine	-3.564	2.02	0.009	down

## Rescue prednisolone treatment for decompensated cirrhosis in autoimmune hepatitis: A case series analysis of clinical features and outcomes

**Lin Han^1^, Shuhong Liu^2^, Zheng Dong^1^, Qingsheng Liang^1^, Huan Xie^1^, Xiaolong Lu^3^, Ying Sun^1^**

^1^Department of Hepatology, the Fifth Medical Center of PLA General Hospital, Beijing, China;

^2^Department of Pathology and Hepatology, the Fifth Medical Center of PLA General Hospital, Beijing, China;

^3^PLA Medical School, Beijing, China

Address for Correspondence: Ying Sun, Email: sunying_302@163.com

### Abstract

**Background**: Autoimmune hepatitis (AIH) is an immune-mediated liver disorder characterized by progressive hepatocellular inflammation and injury. Up to one-third of patients present with cirrhosis at diagnosis. Progression to decompensated cirrhosis (AIH-DC) leads to worsening liver function and serious complications, resulting in poor prognosis. While glucocorticoid therapy remains the cornerstone of AIH management and can achieve a biochemical response rate of 62.5% even in cirrhotic patients, its use in AIH-DC is challenging due to an increased risk of adverse events attributable to hypersplenism, hypoalbuminemia, hyperbilirubinemia, and portosystemic shunting. This study aimed to evaluate individualized prednisolone regimens and adverse event monitoring strategies in AIH-DC patients. **Methods**: A retrospective analysis was conducted on six AIH-DC patients who received first-line prednisolone monotherapy between January 2022 and December 2023, among whom four had acute-on-chronic liver failure (ACLF) at onset. All patients met the simplified AIH diagnostic criteria (score ≥6). Data collected included: (1) baseline demographics, comorbidities, laboratory results, decompensation events, imaging, and histopathology where available; (2) treatment details including initial prednisolone dose and tapering schedule; and (3) follow-up data on laboratory values, Child-Pugh and model for end-stage liver disease (MELD) scores, imaging/histopathological changes, resolution of complications, and adverse events. **Results**: All six cases were female, with an age range of 37-70 years. Three had confirmatory liver histopathology. Comorbidities: one with chronic obstructive pulmonary disease (COPD), one with COPD and cardiac/renal disease. Baseline complications: five with mild-to-moderate ascites, two with esophageal varices, two with spontaneous bacterial peritonitis (SBP). The baseline Child-Pugh score ranged from 6 to 13 points, and the MELD score ranged from 11 to 26 points. Prednisolone regimens: three cases with MELD <25 received` an initial intravenous dose of 40 mg methylprednisolone (equivalent to 50 mg prednisolone), one of them starting on 30 mg oral prednisolone before admission and increasing to 40 mg intravenous methylprednisolone due to poor control. One case with MELD 26 and recurrent infections received an initial dose of 20 mg. These four maintained 20 mg or above for 8-24 weeks. Additionally, two cases received an initial dose of 10 mg: one elderly case with MELD 17 and recurrent pneumonia, and another with MELD 7, poor hepatic reserve function, and spontaneous bacterial peritonitis (SBP). The total duration of therapy ranges from 67 to 153 weeks, and the minimum maintenance dose ranges from 1.25 to 10 mg. Follow-up: two achieved complete biochemical response at 6 months (CBR-6), four achieved CBR-12. All had Child-Pugh 5 at endpoint. Imaging showed improved liver morphology and spleen size. Two cases had repeat biopsy at week 120: hepatic inflammation nearly resolved, fibrosis reduced by 1-2 stages. Ascites resolved in all by weeks 8-136. Adverse events (first 3 months): three with transient ascites exacerbation, one with Severe ascites (resolved after 1 year), one with grade 2 hepatic encephalopathy, one with pneumonia and urinary tract infection (UTI). No life-threatening events. **Conclusions**: For AIH-DC patients, individualized prednisolone monotherapy can achieve favorable efficacy. Cirrhosis- and glucocorticoid-related adverse events are prone to occur within 3 months of treatment, requiring close monitoring and timely intervention. Moreover, AIH-DC requires a relatively long time to achieve CBR, so low-dose maintenance therapy must be maintained. This study provides practical experience for managing such complex cases.

**How to cite this abstract**: Han L, Liu S, Dong Z, Liang Q, Xie H, Lu X, *et al*. Rescue prednisolone treatment for decompensated cirrhosis in autoimmune hepatitis: A case series analysis of clinical features and outcomes. J Transl Intern Med 2025; 13: A643-A646.

**Table 1:** Baseline characteristics and treatment details of 6 cases with decompensated autoimmune cirrhosis

Characteristics	Case 1	Case 2	Case 3	Case 4	Case 5	Case 6
Age(years)	49	40	43	37	70	55
Gender	Female	Female	Female	Female	Female	Female
BMI	29.38	23.63	24.77	30.85	24.56	26.95
Comorbidity	None	None	None	COPD	COPD, Cardiac Pacemaker Implantation Status, CKD	None
Alcohol Consumption History	No	No	No	Yes	No	No
Time from Initial Onset to Decompensated Cirrhosis(weeks)	3.0	6.0	48.1	56.6	451.3	145.6
Time from Decompensated Cirrhosis Onset to Treatment(weeks)	0.6	2.7	3.1	1.7	0.0	17.9
Number of Disease Recurrences Before Treatment	0	0	3	3	7	5
Decompensated Cirrhosis Complications Before Treatment	Mild Ascites, MEV	Mild Ascites	Moderate Ascites, SBP	SEV	Mild Ascites	Moderate Ascites, SBP
High-Risk Prednisolone-Associated Events Before Treatment	ACLF	ACLF	ACLF, Gastrointestinal Ulcer, SBP, Oral Candidiasis	ACLF, SEV, COPD	Advanced Age, Recurrent Infections, Multiple Comorbidities	SBP
Baseline Indices						
White Blood Cell(×109/L)	3.1	5.5	11.2	3.8	4.4	4.1
Platelet(×109/L)	79.0	119.0	166.0	112.0	116.0	103.0
International Normalized Ratio	1.7	2.2	2.2	1.89	1.2	1.5
Total Bilirubin(μmol/L)	127.5	157.0	263.5	285.1	36.0	16.6
Alanine Aminotransferase(U/L)	233.0	666.0	73.0	515	610.0	30.0
Aspartate Aminotransferase(U/L)	377.0	824.0	175.0	885	438.0	47.0
Alkaline Phosphatase(U/L)	207.0	178.0	155.0	279.0	179.0	88.0
γ-Glutamyl Transferase(U/L)	318.0	214.0	35.0	37.0	61.0	29.0
Albumin(g/L)	21.0	26.0	26.0	32.0	37.0	20.0
Prealbumin(mg/L)	16.0	23.0	35.0	19.0	77.0	46.0
Cholinesterase(U/L)	1961.0	4629.0	1538.0	2739.0	3354.0	1827.0
Creatinine(μmol/L)	60.0	75.0	85.0	47.0	134.0	49.0
Immunoglobulin G(g/L)	32.1	23.6	25.7	21.75	26.6	20.5
Positive Autoantibodies	anti-LC-1, pANCA	ANA	ANA, SMA	ANA, SMA	ANA, SMA	ANA
Child-Pugh Score	11	10	13	10	6	7
MELD Score	20	24	26	24	17	11
Liver Biopsy	Yes	Yes	No	No	Yes	No
Simplified Diagnostic Criteria Score for AIH	8	8	6	6	8	6
Prednisolone Initial Dose(mg)	50	50	20	50	10	10
Prednisolone Maintenance Duration(20mg and above, weeks)	24	10	8	11	-	-
Total Prednisolone Maintenance Duration(weeks)	122	123	153	67	106	136
New Complications Within 12 Weeks of Treatment	None	None	Moderate Ascites	Moderate Ascites, HE(Stage 2), Transient Fever	Mild Ascites Pneumonia, Urinary Tract Infections	Severe Ascites
Treatment to Ascites Resolution Time(weeks)	12	12	8	8	8	136
Child-Pugh Score after 48 weeks of treatment	5	5	5	5	5	8
MELD Score after 48 weeks of treatment	6	6	6	11	4	5
Child-Pugh Score at Last Follow-Up	5	5	5	5	5	5
MELD Score at Last Follow-Up	1	6	5	11	3	5

BMI, body mass index; AIH, autoimmune hepatitis; COPD, chronic obstructive pulmonary disease; CKD, chronic kidney disease; SBP, spontaneous bacterial peritonitis; MEV, mild esophageal varices; SEV, severe esophageal varices; ACLF, acute-on-chronic liver failure; anti-LC-1, anti-liver cytosol antibody type 1; pANCA, perinuclear anti-neutrophil cytoplasmic antibody; ANA, anti-nuclear antibody; SMA, anti-smooth muscle antibody; MELD, model for end-stage liver disease; HE, hepatic encephalopathy.

**Figure:**
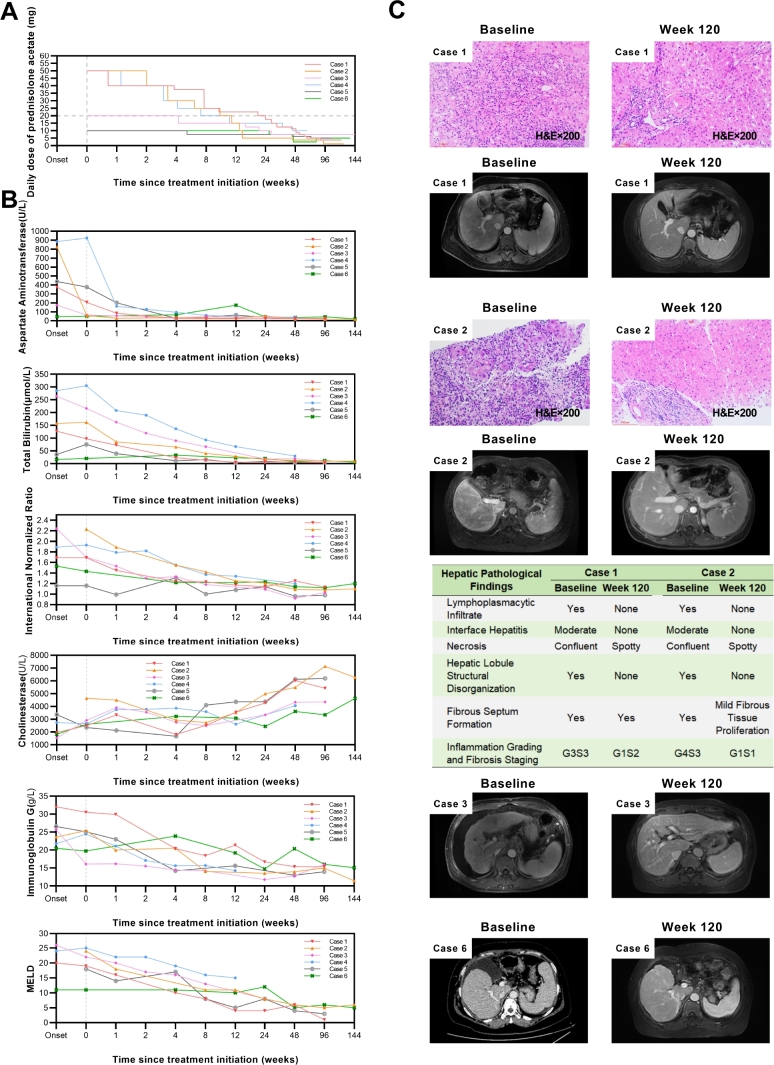


## Quantitative proteomic analysis of oxaliplatin induced peripheral neurotoxicity

**Yanting Zhou, Hansong Yu, Xiaoxiao Wang, Yuxia Wu, Yuetong Pan, Liling Wen, Tianjing Tang, Xincheng Zhuang, Xiang Li, Yichen Li, Hongyan Li**

General Surgery Department, Xuanwu Hospital, Beijing, China

Address for Correspondence: Hongyan Li, Email hongyanli@xwhosp.org

### Abstract

**Background**: Oxaliplatin (OXA) is a third-generation platinum-based chemotherapeutic agent that has become a standard component of first-line therapy for colorectal and other gastrointestinal cancers. Compared with earlier platinum derivatives, OXA causes less nephrotoxicity and ototoxicity. However, oxaliplatin-induced peripheral neurotoxicity (OIPN) remains a frequent and dose-limiting adverse reaction. Acute cold-sensitive neuropathy occurs in most patients shortly after treatment, while chronic progressive neuropathy severely impairs motor coordination, daily activities, and long-term quality of life. Current clinical management relies mainly on dose adjustment, as effective preventive or therapeutic agents are lacking, and the precise molecular mechanisms remain unclear. **Methods**: To comprehensively characterize proteome alterations associated with OIPN, we established mouse models of short-term and long-term OXA exposure and conducted a quantitative proteomic analysis of dorsal root ganglion (DRG) tissues using a data-independent acquisition (DIA)-based mass spectrometry strategy. Differentially expressed proteins were clustered and subjected to functional annotation, Kyoto Encyclopedia of Genes and Genomes (KEGG) enrichment, and gene set variation analysis to identify perturbed biological processes. Furthermore, the therapeutic potential of diroximel fumarate (DRF), an FDA-approved oral fumarate indicated for multiple sclerosis with known antioxidative properties, was evaluated in vivo using behavioral assays, immunohistochemistry of intraepidermal nerve fibers, and western blot validation of key proteins. **Results**: Proteomic profiling revealed 1128 differentially regulated proteins across treatment groups, which were organized into six temporal clusters. Functional enrichment analyses demonstrated that OXA administration induced profound disturbances in cellular metabolism, particularly glycolysis and amino acid metabolism, as well as glutathione-mediated redox pathways. Significant changes were observed in ion channels, including upregulation of potassium (Kv1.1/Kv1.2) and calcium channels (CACNB1, CACNA2D1), and abnormal regulation of chloride channels (CLIC1, CLCN2), implicating neuronal hyperexcitability in OIPN pathogenesis. Mitochondrial dysfunction was evidenced by downregulation of cytochrome c oxidase subunits (NDUFA4, COX4I1) and voltage-dependent anion channels (VDAC2/3), suggesting impaired ATP production and excessive ROS generation. In response, multiple oxidoreductases (GPX3, GSTM2, GSTM5, NQO1) were upregulated, reflecting compensatory activation of antioxidant defenses. Importantly, DRF administration significantly alleviated neuropathic symptoms, increased intraepidermal nerve fiber density, restored mitochondrial protein expression, and attenuated oxidative stress responses, thereby confirming its neuroprotective role. **Conclusions**: This study provides a systematic proteomic map of OXA-induced peripheral neurotoxicity and identifies key molecular networks involved in its development. The data highlight the interplay of metabolic reprogramming, ion channel dysregulation, mitochondrial damage, and oxidative stress as major drivers of OIPN. Moreover, our findings provide the first preclinical evidence supporting the repositioning of DRF as a potential therapeutic option to mitigate OIPN. Collectively, these insights deepen mechanistic understanding of chemotherapy-induced neuropathy and may inform the development of targeted strategies to improve patient outcomes during oxaliplatin-based treatment.

**How to cite this abstract**: Zhou Y, Yu H, Wang X, Wu Y, Pan Y, Wen L, *et al*. Quantitative proteomic analysis of oxaliplatin induced peripheral neurotoxicity. J Transl Intern Med 2025; 13: A647-A648.

**Figure 1:**
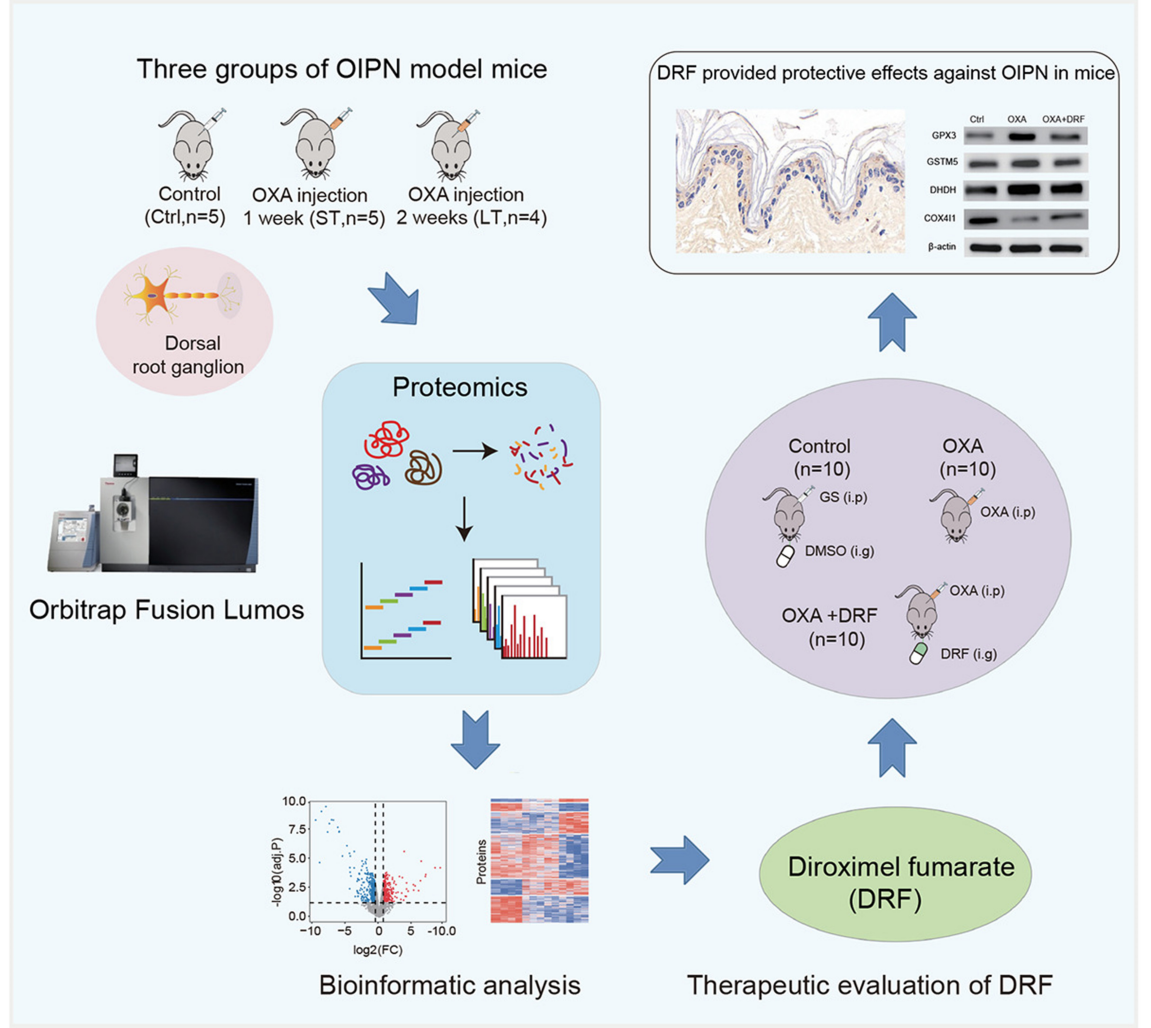
Workflow of the quantitative proteomic analysis and administration of diroximel fumarate (DRF) alleviated OIPN in mice.

## Mechanism of coumainr in the anti-liver cancer activity by promoting NK cell activation in *Sophora tonkinensis*

**Jincai Wen^1,2,3^, Xiaoyan Zhan^1,2^, Xiaohe Xiao^1,2,3^, Zhaofang Bai^1,2,3^**

^1^Department of Hepatology, The Fifth Medical Center of Chinese PLA General Hospital, Beijing, China;

^2^China Military Institute of Chinese Materia, Fifth Medical Center of Chinese PLA General Hospital, Beijing, China;

^3^School of Pharmacy, Chengdu University of Traditional Chinese Medicine, Chengdu, Sichuan Province, China

Address for Correspondence: Xiaohe Xiao, Email: pharmacy_302@126.com; Zhaofang Bai, Email: Baizf2008@hotmail.com

### Abstract

**Objective**: The present study aims to systematically elucidate the specific molecular mechanisms by which coumarin from Sida root exerts its anti-liver cancer effects through the promotion of natural killer (NK) cell activation. The focus is on investigating whether coumarin can effectively inhibit the progression of liver cancer by enhancing the key effector functions of NK cells, including cytokine secretion and expression of cytotoxic proteins. Furthermore, this study seeks to provide novel intervention strategies and potential targets for liver cancer immunotherapy based on natural products. **Materials and Methods**: An *in vitro* cell experimental system was employed to screen the effects of various active components from Sida root on the functions of NK-92 cells. The secretion levels of effector cytokines (such as IFN-γ) in the cell culture supernatant were detected using ELISA kits. The protein expression and mRNA transcription levels of related effector proteins (such as granzyme B and perforin) were detected using Western blotting and quantitative real-time PCR (qPCR) techniques, respectively. The expression characteristics of NK cell surface receptors and markers were analyzed by flow cytometry. Subsequently, the functional effects of the target component (Coumarin) were verified in primary human NK cells, with a focus on evaluating its regulatory effects on the NK cell surface receptor profile and functional markers. Furthermore, an H22 hepatocellular carcinoma subcutaneous xenograft mouse model was constructed to systematically evaluate the inhibitory effects of Coumarin on tumor growth. Tumor volume changes were dynamically monitored during the experimental period. At the end of the experiment, the infiltration ratios of immune cells (such as NK cells and T cell subsets) in the spleen and tumor tissues of mice were detected by flow cytometry, and the expression characteristics of immune-related genes were analyzed using qPCR technology to comprehensively elucidate the regulatory effects of Coumarin on the immune landscape of the tumor microenvironment. **Results**: The results of the study demonstrated that Coumarin from Sida root significantly promotes the activation of NK cells. Under the synergistic action of IL-12, Coumarin not only upregulates the transcription levels of IFN-γ, granzyme B, and perforin in NK-92 cells (as evidenced by significant increases in mRNA expression detected by qPCR), but also enhances their secretion functions (confirmed by increased cytokine secretion levels detected by ELISA and elevated protein expression of granzyme B and perforin shown by flow cytometry). Consistent promoting effects were observed in primary human NK cells. *In vivo* experiments further revealed that Coumarin effectively inhibits tumor growth in the H22 hepatocellular carcinoma model (with significant reduction in tumor volume). Flow cytometry analysis showed that Coumarin significantly increases the infiltration ratios of immune cells, such as NK cells, in both tumor and spleen tissues of mice. These findings suggest that Coumarin not only directly inhibits tumor growth by enhancing NK cell functions but also significantly elevates the infiltration levels of immune cells in the tumor microenvironment, thereby reshaping the antitumor immune microenvironment. **Conclusion**: Coumarin from Sida root significantly promotes NK cell activation through the synergistic action of IL-12, characterized by increased IFN-γ secretion and upregulated expression of key cytotoxic proteins such as granzyme B and perforin. In the H22 hepatocellular carcinoma model, Coumarin effectively inhibits tumor growth by enhancing NK cell functions and increasing the infiltration ratios of immune cells (NK cells, CD4^+^ T cells, and CD8^+^ T cells) in the tumor microenvironment, thereby exerting its immunomodulatory antitumor effects. This study elucidates the immunological mechanisms underlying the antitumor activity of Coumarin as an active component from Sida root and provides experimental evidence for its potential clinical translation.

**How to cite this abstract**: Wen J, Zhan X, Xiao X, Bai Z. Mechanism of coumainr in the anti-liver cancer activity by promoting NK cell activation in *Sophora tonkinensis*. J Transl Intern Med 2025; 13: A649.

## MGMT expression correlated with synergistic action of temozolomide and PARP inhibitor

**Yanxin Lu, Yiqiang Zhou, Wentao Wang, Xupeng Yue**

Zunyi Medical University Zhuhai Campus, Department of Neurosurgery, Xuanwu Hospital, Capital Medical University, Center for Cancer Research

Address for Correspondence: Yanxin Lu, Email: yanxinlu@zmu.edu.cn

### Abstract

**INTRODUCTION**: Temozolomide (TMZ) is an alkylating agent currently used as first-line therapy for patients with glioblastoma (GBM) due to its DNA-damaging effect. However, almost half of GBMs are TMZ resistant due to expression of O6-methyguanine-DNA-methytransferase (MGMT), which counteracts the TMZ induced O6-methyguanine. Although MGMT inhibitors such as lomeguatrib (LOM) and O6-BG have demonstrated in vitro efficacy in enhancing DNA damage and promoting apoptosis, their clinical development has been hindered by significant toxicity and uncertain therapeutic benefits. Therefore, MGMT remains a promising target for glioma therapy, and the discovery and development of effective MGMT inhibitors may offer novel therapeutic strategies for the treatment of glioma. **METHODS**: We investigated the chemo-resisitance and DNA damage level in MGMT negative and positive *IDH1*x (wild type and mutated IDH1) glioma cells. Further, we attempted to enhance chemo-senstivity in MGMT positive *IDH1*x cells by suppressing MGMT DNA repair activity using PARP inhibitor Olaparib. **RESULTS**: In our previously study, MGMT potentiate the chemo-resistance of *IDH1*x cells through decrease DNA damage; PARP is require for MGMT mediated resistance to TMZ, we found the inhibitor of PARP has double effect activity. The results showed that blocking PARP activity could significantly improve the sensitivity of MGMT positive glioma cells to TMZ and significantly elongate the survival of PDX animal models. Further studies showed that pADPR, which represent the activation level of Poly ADP-ribose polymerase (PARP) DNA repair pathway, is increased in MGMT positive cells under TMZ treatment. After blocking PARP activity, the level of cell DNA damage and apoptosis was significantly increased, the cell viability was decreased. These results suggest that PARP may be involved in MGMT related O6-MeG DNA repair pathway, and there may be an interaction between MGMT and PARP. The Co-IP confirmed the interaction between MGMT and PARP. **CONCLUSION**: This study revealed the cross-talk relationship between PARP and MGMT in the repair process of O6-MeG, elucidated the molecular mechanism of PARP inhibitor reversing MGMT-positive glioma drug resistance, and provided novel therapeutic strategies for clinical treatment of chemo-resistant glioma.

**How to cite this abstract**: Lu Y, Zhou Y, Wang W, Yue X. MGMT expression correlated with synergistic action of temozolomide and PARP inhibitor. J Transl Intern Med 2025; 13: A650.

## Efficacy and safety of pembrolizumab and lenvatinib in combination with GEMOX *via* different administration routes in advanced intrahepatic cholangiocarcinoma: A retrospective analysis of real-world evidence

**Hongli Yu, Caiyun Peng, Shuai Wang, Yinying Lu, Jiamin Cheng**

Comprehensive Liver Cancer Center, The Fifth Medical Center of PLA General Hospital, Beijing, China

Address for Correspondence: Jiamin Cheng, Email: chengjiamin300@163.com

### Abstract

**Background**: The combination of programmed death (PD)-1 inhibitors with tyrosine kinase inhibitors (TKIs) and GEMOX (Gemcitabine+Oxaliplatin) has shown potential in the treatment of advanced intrahepatic cholangiocarcinoma (ICC). However, the impact of different administration routes of GEMOX, specifically hepatic artery infusion chemotherapy (HAIC) and intravenous (IV) injection, on the efficacy and safety of this regimen remains unclear. **Methods**: It was a retrospective study enrolling patients with advanced ICC who received pembrolizumab and lenvatinib combined with GEMOX via either HAIC or intravenous injection. Eligibility criteria included pathologically confirmed ICC. Patients in the HAIC group received the drugs through a hepatic artery catheter, while those in the intravenous group received the standard intravenous infusion. The primary endpoint was overall survival (OS), and secondary endpoints included progression-free survival (PFS), objective response rate (ORR), disease control rate (DCR), which were evaluated in accordance with the mRECIST criteria. Log-rank tests were employed to compare the survival data. Adverse events (AEs) were recorded according to the CTCAE v5.0. **Results**: Seventy-one patients were included in the analysis, with 37 patients in the HAIC group and 34 patients in the IV group. There were no significant differences in gender, age, tumor stage, and performance status (PS) score between the two groups. The median follow-up time was 8.8 months. The estimated median OS in the HAIC group was 27.9 (95%CI: 16.7-39.2) months, while in the IV group it was 12.4 (95%CI: 9.9-14.9) months (*P* = 0.026). For PFS, the median PFS in the HAIC group was 7.1 (95%CI: 5.3-8.9) months and in the IV group was 8.5 (95%CI: 5.1-11.8) months (*P* = 0.743). In the HAIC group, the ORR was 35.1%, and the DCR was 81.1%. In the IV group, the ORR was 41.2%, and the DCR was 76.5%. Regarding safety, the incidence of grade 3/4 AEs was 29.7% (11/37) in the HAIC group and 35.3% (12/34) in the IV group. **Conclusions**: In the treatment of advanced intrahepatic cholangiocarcinoma with the combination of pembrolizumab, lenvatinib, and GEMOX, the HAIC route for delivering GEMOX may offer a survival advantage.

**How to cite this abstract**: Yu H, Peng C, Wang S, Lu Y, Cheng J. Efficacy and safety of pembrolizumab and lenvatinib in combination with GEMOX *via* different administration routes in advanced intrahepatic cholangiocarcinoma: A retrospective analysis of real-world evidence. J Transl Intern Med 2025; 13: A651.

**Table 1:** Outcomes

Outcomes	HAIC group (*N* = 37)	IV group (*N* = 34)	*P* value
Tumor response			
CR	0	1(2.9%)	
PR	13 (35.1%)	13 (38.2%)	
SD	17 (45.9%)	12(35.3%)	
PD	7 (18.9%)	8(23.5%)	
ORR	35.1%	41.2%	
DCR	81.1%	76.5%	
OS (median, 95%CI)	27.9 (16.7-39.2)	12.4 (9.9-14.9)	0.026
PFS (median, 95%CI)	7.1 (5.3-8.9)	8.5 (5.1-11.8)	0.743

HAIC, hepatic artery infusion chemotherapy; IV, intravenous; CR, complete response; PR, partial response; SD, stable disease; PD, progressive disease; ORR, objective response rate; DCR, disease control rate; OS, overall survival, PFS, progression-free survival.

